# Transcription Factors in Fungi: TFome Dynamics, Three Major Families, and Dual-Specificity TFs

**DOI:** 10.3389/fgene.2017.00053

**Published:** 2017-05-04

**Authors:** Ekaterina Shelest

**Affiliations:** Systems biology/Bioinformatics group, Leibniz Institute for Natural Product Research and Infection Biology, Hans Knoell InstituteJena, Germany

**Keywords:** transcription factors, TFome, fungal genomes, gene family expansions, Zn2 Cys6 Zn cluster TF, DNA-binding domains

## Abstract

Transcription factors (TFs) are essential regulators of gene expression in a cell; the entire repertoire of TFs (TFome) of a species reflects its regulatory potential and the evolutionary history of the regulatory mechanisms. In this work, I give an overview of fungal TFs, analyze TFome dynamics, and discuss TF families and types of particular interest. Whole-genome annotation of TFs in more than 200 fungal species revealed ~80 families of TFs that are typically found in fungi. Almost half of the considered genomes belonged to basidiomycetes and zygomycetes, which have been underrepresented in earlier annotations due to dearth of sequenced genomes. The TFomes were analyzed in terms of expansion strategies genome- and lineage-wise. Generally, TFomes are known to correlate with genome size; but what happens to particular families when a TFome is expanding? By dissecting TFomes into single families and estimating the impact of each of them, I show that in fungi the TFome increment is largely limited to three families (C6 Zn clusters, C2H2-like Zn fingers, and homeodomain-like). To see whether this is a fungal peculiarity or a ubiquitous eukaryotic feature, I also analyzed metazoan TFomes, where I observed a similar trend (limited number of TFome-shaping families) but also some important differences connected mostly with the increased complexity in animals. The expansion strategies of TF families are lineage-specific; I demonstrate how the patterns of the TF families' distributions, designated as “TF signatures,” can be used as a taxonomic feature, e.g., for allocation of uncertain phyla. In addition, both fungal and metazoan genomes contain an intriguing type of TFs. While usually TFs have a single DNA-binding domain, these TFs possess two (or more) different DNA-binding specificities. I demonstrate that dual-specific TFs comprising various combinations of all major TF families are a typical feature of fungal and animal genomes and have an interesting evolutionary history involving gene duplications and domain losses.

## Introduction

Transcription factors play a major role in gene expression coordination. The TF cohort defines the regulatory capacity of an organism, and the evolutionary history of TF families reflects the history of the cognate regulatory mechanisms; the analysis of the TF repertoire is therefore instructive from both the functional and evolutionary points of view.

In 2008, I reviewed the predicted occurrences of DNA-binding domains in the then available 62 fungal genomes, which revealed a set of 37 “fungal” TF families (Shelest, 2008). Eight years later, a significantly larger number of fungal genomes is available; moreover, the sequencing effort has been distributed more evenly across the fungal phyla, providing additional data for earlier underrepresented basidio- and zygomycetes (a group of basal fungi including the phyla Mucoromycota and Zoopagomycota; Spatafora et al., 2016). This led me to revise the previous analysis; furthermore, some questions could not be answered and even did not arise at times when we could not access such diverse and abundant data.

The eukaryotic genome size varies through a couple of orders of magnitude. Whole sets of species' TFs, often referred as TFomes, generally follow the increase in genomic size, and in eukaryotes their fraction in the proteome is more or less constant. It has been already shown that the number of TF genes correlates with the number of protein-coding genes following a power law (Iyer et al., 2008; Charoensawan et al., 2010a). It has been also shown that while in bacteria the exponent is close to quadratic, in eukaryotes it is lower (around 1.3; van Nimwegen, 2003; Babu et al., 2004; Aravind et al., 2005; Charoensawan et al., 2010a). As demonstrated by Charoensawan et al. (2010a), the changes in number of distinct families cannot explain the overall TFome growth, hence the TFs' number increases mainly through gene duplication of existing families.

All the accepted rules of TFome growth are fully applicable to fungal genomes (de Mendoza et al., 2013; Todd et al., 2014). However, in all these excellent studies the TFomes were considered as a whole. I thought that it would be pertinent to ask the following question: *how* do transcription factor numbers increase? Do all TF families expand uniformly or maybe some of them give a larger contribution to the overall TFome expansion? Here I provide an analysis of differential TFome dynamics, and show the primordial role of three main TF gene families: Zn clusters, C2H2 Zn fingers, and homeodomain (HD)-like. Moreover, similar trends, i.e., a limited number of families responsible for the TFome growth, are observed for other eukaryotes, which is shown here on the examples of animal TFomes.

Another aspect of the TF family distribution regards lineage-specific expansions and consequent differences in relative portions of TF families in TFomes. Significant frequency differences have been shown for particular families in various eukaryotic lineages (Charoensawan et al., 2010b; de Mendoza et al., 2013; Thiriet-Rupert et al., 2016) and specifically in two fungal phyla, Ascomycota and Basidiomycota (Todd et al., 2014). These observations are confirmed by the present study for a larger set of genomes, including some of animals and protists. I try to demonstrate that these differences can be used as taxonomic features, which can be especially helpful for fine-tuning phyla with uncertain taxonomic position. I introduce a notion of a TF signature, a lineage-specific pattern of distribution of representative TF families, and show how it can be applied to solving some taxonomy-related problems.

Finally, I would like to discuss a particular type of TF gene families that are found in fungi and also in metazoa and plants: factors with two (or more) different DNA-binding specificities (dual-specificity TFs). The fact that there can be more than one different DNA-binding domains (DBDs) in a TF has been noticed before (e.g., Aravind and Koonin, 1999; Tsuji et al., 2000; Charoensawan et al., 2010b). Most of the functionally characterized DBD combinations contain an HTH (helix-turn-helix) counterpart, in particular homeodomains (HD; Aravind and Koonin, 1999; Khare et al., 2004; see Aravind et al., 2005 for a short discussion). Combinations of homeodomains with Zn fingers (both of specific type, ZF_HD) are described and represented by a separate entry in databases (e.g., PF04770 in PFAM; IPR006456 in InterPro); homeobox-leucine zipper genes are plant-specific (Schena and Davis, 1994); combination of CUT domain with HDs has been described in detail by Lannoy et al. (1998). So far, other classes of dual-specific TFs are much less known. In 2000, Tsuji et al investigated regulators of melanin biosynthesis from *Colletotrichum lagenarium* and *Magnaporthe grisea* (Cmr1p and Pig1p, respectively), which were the first TFs described containing both C2H2 Zn finger and C6 Zn cluster DNA binding motifs. Deletion analysis of Cmr1p showed that both domains were distinctly functional *in vivo*: the Zn cluster deletion led to complete loss of melanin production, whereas deletion of the C2H2 counterpart only reduced it. Several further homologs of Cmr1 (Kihara et al., 2008; Cho et al., 2012) or TFs with similar domain structure (e.g., Zhang et al., 2004) were described in literature but the functionality of the domains was not investigated. On the whole-genome scale, little is known about dual-specific TFs. In this work, I show that dual-specificity TFs with combinations of all major TF families are typical for fungal and animal genomes. Furthermore, I investigate evolutionary relationships in a group of paralogous dual-specific TFs in ascomycete fungus *Aspergillus nudulans* and demonstrate that the family has undergone a series of duplications accompanied by quite intensive loss of the second binding specificity. This brief study supports the idea that dual-specificity TFs are extremely interesting from functional and evolutional perspectives and definitely deserve a profound analysis with experimental characterization.

## Results

### Transcription factors in fungi—revisited

Genome-wide DNA-binding domain predictions detect 122 transcription factor-type DBD families (in the following TFDFs) in the sequenced fungal and microsporidial genomes (Table S1, see Section Methods). Some families that appear sporadically (<5 species) and are represented by a limited numbers of genes, may trivially represent erroneous annotation or even genome contamination, however, as many of those scantily represented families are of bacterial or viral origin they may be relevant by representing instances of recent horizontal gene transfer (HGT). Indeed, of 42 marginally represented families, 28 (67%) are bacteria-, archea- or virus-specific, 11 are metazoan, and 3 are plant-specific (Table S2A). Although these TFs deserve further investigation in terms of their origin and function, I will not consider them in the further analysis.

After removing the marginal families from the list, we get 80 TFDFs that are typically found in fungal genomes (Table 1; Table S3). The great majority of them are shared with either prokaryotes, or other eukaryotes, or both (Table S2B). In accordance with previous observations (Shelest, 2008; Todd et al., 2014) three TFDFs: APSES, Mating-type MAT α1, and Copper fist DBD are fungal-specific, i.e., they are found exclusively in fungal genomes. The Zn cluster family, which usually is referred as a typical fungal-specific family, has been actually detected in various non-fungal species; this patchy distribution has been already discussed by several authors (e.g., Weirauch and Hughes, 2011; Scazzocchio, 2014) but there is no commonly accepted opinion on how they evolved. One possible scenario could be that Zn clusters are a fungal-specific family that was born at the onset of fungal radiation and afterwards underwent numerous independent sporadic HTGs to other lineages. The other scenario assumes that it is a very ancient eukaryotic family massively lost in most of lineages but having come to prosper in fungi. Which of the scenarios was realized, remains an open question, which possibly can be answered after inspection of more eukaryotic genomes. On the other hand, Zn clusters are specific to fungi in that sense that they have been detected in absolutely all fungal species analyzed so far. This makes Zn clusters a “necessary but not sufficient” fungal feature: a species can be assigned to the fungal kingdom only if it has a Zn cluster in its genome but an occurrence of a Zn cluster does not alone provide a proof of being a fungus.

**Table 1:** **TF-type DNA-binding domains typically found in fungal species**.

**IPR ID**	**DBD Name**
IPR000005	Helix-turn-helix, AraC type
IPR000007	Tubby, C-terminal
IPR000197	Zinc finger, TAZ-type
IPR000232	Heat shock factor (HSF)-type, DNA-binding
IPR000327	POU-specific
IPR000418	Ets
IPR000551	Bacterial regulatory protein, MerR
IPR000571	Zinc finger CCCH-type
IPR000679	Zinc finger, GATA-type
IPR000792	Bacterial regulatory protein, LuxR
IPR000814	TATA-box binding
IPR000818	TEA/ATTS
IPR000835	Bacterial regulatory protein, MarR
IPR000843	Bacterial regulatory protein, LacI
IPR000944	Transcriptional regulator, Rrf2
IPR000967	Zinc finger, NF-X1-type
IPR001034	Bacterial regulatory protein, DeoR N-terminal
IPR001083	Copper fist DNA-binding^*^
IPR001138	Zn2 Cys6 Zn_cluster^*^
IPR001275	DM DNA-binding
IPR001289	CCAAT-binding TF, subunit B
IPR001356	Homeobox
IPR001387	Helix-turn-helix type 3
IPR001471	Pathogenesis-related TF and ERF, DBD
IPR001523	Paired box protein, N-terminal
IPR001699	Transcription factor, T-box
IPR001766	Fork head transcription factor
IPR001808	Bacterial regulatory protein, Crp
IPR001845	Bacterial regulatory protein, ArsR
IPR001878	Zinc finger, CCHC-type
IPR002059	Cold-shock protein, DNA-binding
IPR002100	Transcription factor, MADS-box
IPR002197	Helix-turn-helix, Fis-type
IPR002653	Zinc finger, A20-type
IPR003150	DNA-binding RFX
IPR003163	APSES-type DNA-binding domain^*^
IPR003316	E2F/dimerisation partner (TDP)
IPR003656	Zinc finger, BED-type predicted
IPR003657	DNA-binding WRKY
IPR003902	Transcriptional regulator, GCM-like
IPR003958	TF CBF/NF-Y/archaeal histone
IPR004022	DDT
IPR004181	Zinc finger, MIZ-type
IPR004198	Zinc finger, C5HC2-type
IPR004333	Transcription factor, SBP-box
IPR004645	DNA-binding protein Tfx
IPR004823	TATA box binding protein associated factor (TAF)
IPR004826	Maf transcription factor
IPR004827	Basic-leucine zipper (bZIP) TF
IPR005011	SART-1 protein
IPR006780	YABBY protein
IPR006856	Mating-type protein MAT alpha 1^*^
IPR007087	Zinc finger, C2H2-type
IPR007196	CCR4-Not complex component, Not1
IPR007396	Negative transcriptional regulator
IPR007604	CP2 transcription factor
IPR007889	Helix-turn-helix, Psq
IPR008895	YL1 nuclear
IPR008917	Eukaryotic transcription factor, Skn-1-like
IPR008967	p53-like transcription factor, DNA-binding
IPR009044	ssDNA-binding transcriptional regulator
IPR009057	Homeodomain-like
IPR009061	Putative DNA binding
IPR009395	GCN5-like 1
IPR010666	Zinc finger, GRF-type
IPR010770	SGT1
IPR010919	SAND-like
IPR010921	Trp repressor/replication initiator
IPR010982	Lambda repressor-like, DNA-binding
IPR010985	Ribbon-helix-helix
IPR011598	Helix-loop-helix DNA-binding
IPR012294	Transcription factor TFIID, C-terminal
IPR013921	TATA-binding related factor
IPR013932	TATA-binding protein interacting (TIP20)
IPR015988	STAT transcription factor, coiled coil
IPR016032	Signal transduction response regulator, C-term. effector
IPR016177	DNA-binding, integrase-type
IPR024061	NDT80 DNA-binding domain
IPR025659	Tubby C-terminal-like domain

* *Fungal-specific families*.

In the fungal kingdom different phyla are phylogenetically quite divergent, thus it will be interesting to investigate if some TFDF are phylum specific. I compared the occurrence of TFDFs in asco-, basidio- and zygomycetes, which revealed some families restricted to one or the other lineage (Figure S1). Interestingly, only one family (IPR006856, Mating-type protein MATα1) is found exclusively in ascomycetes, whereas the more ancient zygomycetes possess four families not shared with other fungi. These families, however, are not unique to zygomycetes and are found in other Eukaryotes and/or in other kingdoms (Table S2). Since zygomycetes (Mucoro- and Zoopagomycota) are the most ancient lineages of fungi, they may preserve some families inherited from LECA (least eukaryotic common ancestor) that are lost in more recent phyla.

### How does the number of TFs grow?—peculiarities of fungal TF gene distributions, and three main families

The number of TF genes is not equal to the number of TF DBDs, because some proteins contain more than one DBD and some domains can be described by more than one DBD model (models can represent families, subfamilies, and superfamilies of domains). In the discussion of TFomes, i.e., entire TF repertoires, we are interested not in the domains but in gene counts, so we switch from consideration of DNA-binding domains to a gene-wise view. To differentiate between TF DBD families and TF gene families, I will refer to the latter as TFgF.

In this study, 115 distinct TF gene types were found in fungal genomes. Some of them, however, were represented only in a very small number of species so they were not considered in the following analysis for the same reasons as stated for the marginal DBDs. These genes types were, however, included in the entire TF repertoire counts. The final number of retained TFgFs was 78 (Table 2; Table S4).

**Table 2:** **Fungal TF gene families**.

**TF Gene family name**
APSES^*^
Bacterial regulatory protein, ArsR
Bacterial regulatory protein, Crp
Bacterial regulatory protein, DeoR N-terminal
Bacterial regulatory protein, LacI
Bacterial regulatory protein, LuxR
Bacterial regulatory protein, MarR
Bacterial regulatory protein, MerR
BESS
bZIP
bZIP + Helix-loop-helix DNA-binding
bZIP + Homeodomain-like
bZIP + C2H2
C2H2/CCHC/CCCH ZF + Homeodomain
C2H2/CCHC/CCCH/C5HC2
CBF/NF-Y/archaeal histone
CCAAT-BindingTF
CCR4-Not complex component, Not1
Cold-shock DBD
Copper fist^*^
Copper fist+Zn finger^*^
DM DNA-binding
DNA-binding protein Tfx
DNA-binding WRKY
DNA-binding, integrase-type
E2F_TDP^b^
Ets
Fork head TF
GATA
GATA + Homeo
GATA + Zn cluster
GCM-like
GCN5L1
Heat shock factor (HSF)-type
HLH, helix-loop-helix
HTH/Homeodomain-like
lambda repressor(-like)/POU
Homeodomain+lambda repressor-like
LexA
MADS-box/SRF
Maf TF
Mating-type protein MATα1^*^^a^
No apical meristem (NAM) protein
p53
Putative DNA binding
Rel homology
RFX_DNA_binding
Ribbon-helix-helix
Rrf2
SAND-like
SART-1
SBP-box
SGT1
Signal transduction response regulator, C-term.
Skn-1
ssDNA-binding transcriptional regulator
STAT
TATA box binding protein associated factor (TAF)
TATA-binding protein interacting (TIP20)
TATA-binding related factor
T-box
TEA/ATTS
TFIID
Trp repressor/replication initiator
Tubby TF
Viral DNA-binding protein
YL1
zf-A20
zf-BED
zf-GRF
zf-MIZ
zf-TAZ
Zn cluster^*^
Zn cluster + bZIP^*^
Zn_cluster+C2H2+Homeodomain^*^^a^
Zn_cluster+Homeodomain
Zn cluster + C2H2/CCHC/CCCH-type Zn fingers^*^
Zn-finger, NF-X1 type

* *Fungal-specific TFgFs (the specificity is assumed based on the involved fungal-specific domains)*.

a *Found exclusively in ascomycetes*.

b *Only in Zygomycetes*.

**Figure 1:**
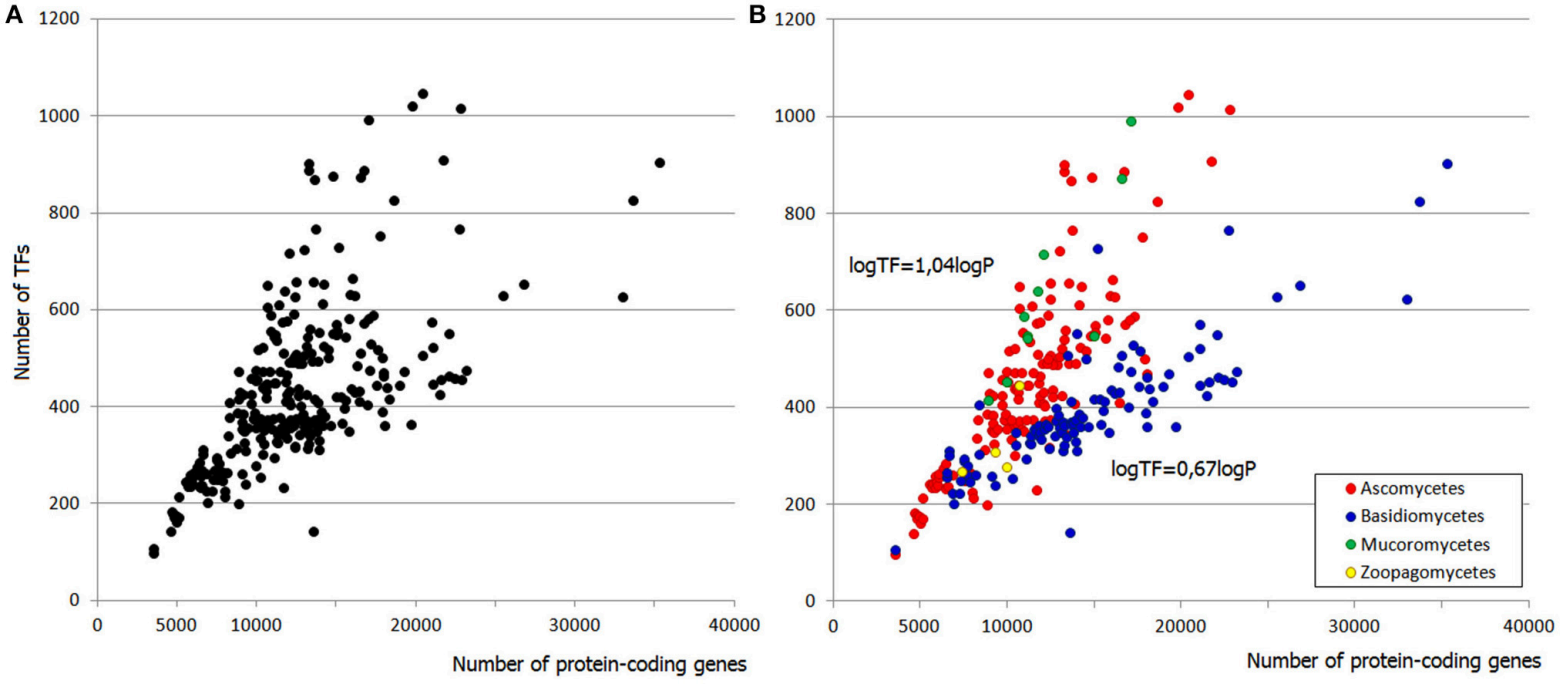
**(A)** Distribution of TF gene families in fungal genomes. **(B)** Two “tails” belong to asco- and basidiomycetes. Each dot represents a species.

Fungal genomes comprise from ~3,000 to ~30,000 protein-coding genes. The proportion of transcription factors in genomes remains practically intact implying that larger genomes have more TFs. This tendency is known from earlier observations (see Section Introduction) and is also confirmed by the present analysis (Figure 1A), which is based on a much larger set of species, including more basidio- and zygomycetes. Regarding the latter, it is worth noting that some of them (e.g., *Rhyzopus oryzae*) are known to have undergone a recent whole genome duplication (WGD; Ma et al., 2009; Corrochano et al., 2016), whereas for other species (e.g., *Lichtheimia corymbifera*) the WGD is under debate (Schwartze et al., 2014). The recent WGD implies quite a different mechanism of TFome growth, so the species with proven WGD history were excluded from the analysis TFome-proteome relationship.

The distribution of TFome sizes in fungal genomes has a “two-tail” shape (Figure 1A), which can hardly be directly approximated by any function but rather seems to be a superposition of two plots. Indeed, this shape is easily explained by considering the main fungal phyla separately: the two tails correspond to asco- and basidiomycetes (Figure 1B). Zygomycetes (which are not monophyletic, therefore mucoro- and zoopagomycetes are considered separately) seemingly fit into the upper tail but they in fact have a larger exponent; however, the number of species is too small to build a reliable model. The distribution in ascomycetes can be approximated by a linear fit (logTF = 1,04logP with *R*^2^ = 0.77; TF stands for the number of TFs, P is the number of protein-coding genes, *R*^2^ is the coefficient of determination), whereas in basidiomycetes it is weaker power-law (logTF = 0.67logP with *R*^2^ = 0.69).

Thus, the total number of TFs correlates with the number of protein-coding genes, but does this equally apply to all TF gene families? In other words, do all families grow proportionally, or do TFomes increase on account of some particular TFgFs?

Some TF gene families are known to be represented in constant numbers in all genomes. Apparently, these TFs, also referred sometimes as “frozen,” do not have any impact on the increment of the total TFs' number. Interestingly, there is a loose connection between a family's growth behavior and its size: many of the “frozen” TFgFs are single- or two-gene families, such as CCAAT-box binding TF, MATα, TATA binding protein, etc. This is not a strict rule, though; but in what follows we will see that family size is a fairly useful indicator of whether or not the family is subject to expansion. For simplicity's sake, I will refer to families in with <5 genes per genome (on average) as “small” and to the others as “abundant.”

**Figure 2:**
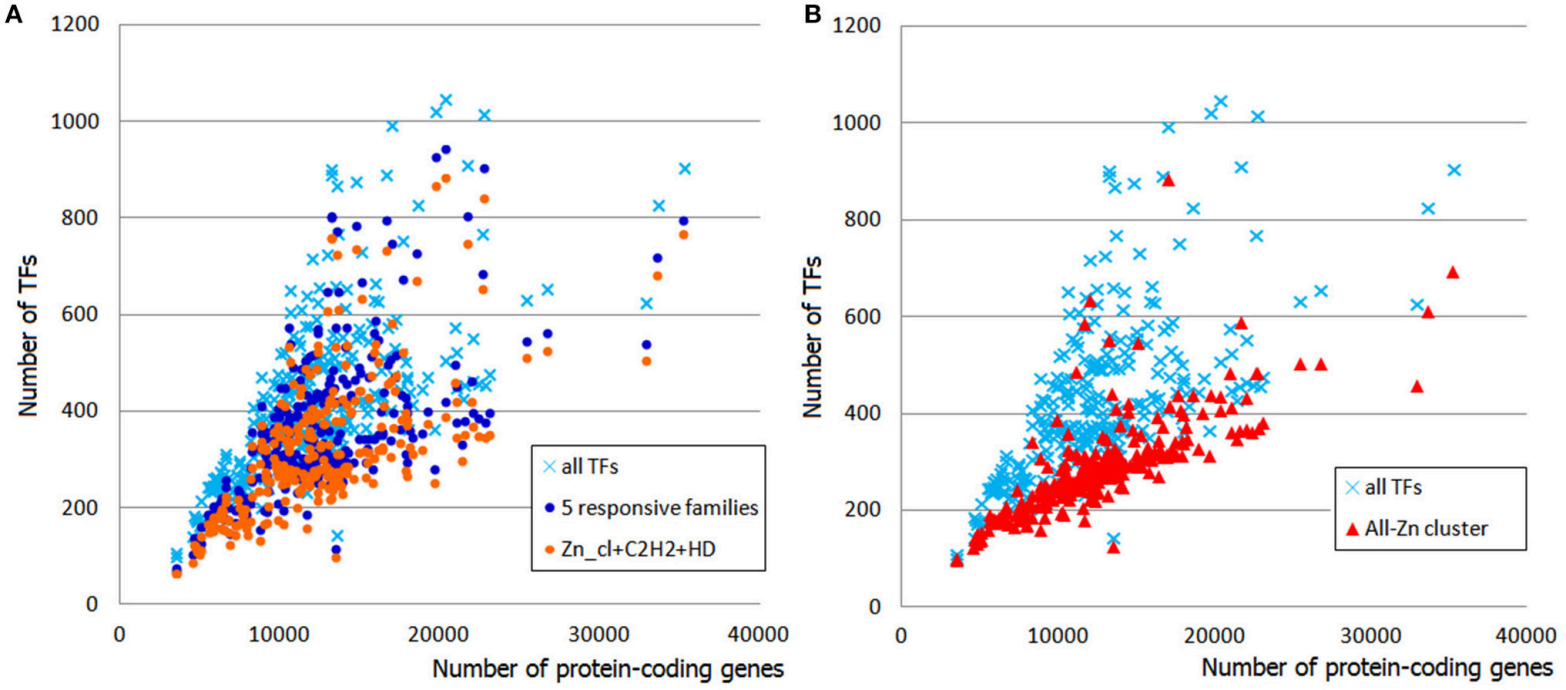
**Contribution of responsive families to TFome expansion in fungal genomes. (A)** The sum of five responsive families (dark blue circles) follows the profile of whole TFomes. The effect can be explained by the input of just three families (Zn cluster, C2H2-like, and HD-like; orange circles). **(B)** The contribution of Zn clusters can be illustrated by subtraction of this family from the whole TFome. Without the Zn cluster family, TFomes are confined to the lower “tail.”

The responsiveness of TFgFs to the proteome size growth was characterized by two parameters: the exponent (*exp*) of the increase and the coefficient of determination *R*^2^ (estimation of the exponent fitting quality; see Section Methods for details). These parameters were calculated for each TF gene family; families that showed the exponent >0.5 were considered as growing with the genome growth and designated as “responsive”; accordingly, the non-growing families (with *exp* < 0.5) were called “non-responsive” (see Section Methods and Table S5). Given the differences between the lineages, the exponent and *R*^2^ were calculated separately for asco- and basidiomycetes. Most of the families in both groups show no growth at all (*exp* = 0) or very low exponents (*exp* < 0.5), which may be considered not significant. Of 78 considered TFgFs, 69 families are “small” and none of them expands in response to genome growth (Table S5). This does not mean that these families are all “frozen”: some show rare and usually peak-shaped species- or lineage-specific expansions (so-called single expansions, see below), which are not correlated to genome size. The negligible input of small TFgFs means, obviously, that the growth of TFomes depends only on the remaining 9 abundant families. Quite unexpectedly, even though the number of abundant families is low, not all of them actually expand. In total there are only five families (Zn cluster, C2H2-like, HD-like, HLH, and bZIP) that respond to the proteome size changes with a significant exponent (>0.5) and coefficient of determination (*R*^2^ > 0.5; Table S5, Figure 2A). In fact just three of them: Zn cluster, C2H2, and HD-like, can alone explain most of the TFome size changes (Figure 2A). It is interesting that the behavior of C2H2 and HD-like does not differ between asco- and basidiomycetes, whereas Zn clusters grow much faster in ascomycetes (the exponent differs by a factor of ~2.5). This largely accounts for the two “tails” seen on the distribution plot: the upper “tail” disappears if we subtract Zn clusters from the total TFs (Figure 2B), so it consists of ascomycete Zn cluster TFs. This observation suggests a particular role and evolutionary history of Zn2 Cys6 Zn clusters. We will return to it in Discussion.

### What about other kingdoms?

It is interesting to ask whether the non-proportional TFgFs increase is a unique fungal feature or it is seen also in other kingdoms. To answer this question, I analyzed TF gene families' occurrences and distributions in 46 metazoan genomes available in the DBD database (http://www.transcriptionfactor.org; Table S6). Not all species with predicted TFs were included in the further analysis of the TFomes. In general, the TFomes' increase analysis makes sense only in monophyletic groups (because we are interested in the growth of the same set of TFs and the TFomes content may be quite different between phylogenetically unrelated groups). The metazoan group is too heterogeneous in this respect, unless we single out phylogenetically coherent groups and consider them separately. The first group would be chordates; the largest coherent group of non-chordate animals available in DBD is Ecdysozoa (Arthropods and Nematodes). A further separation into classes is not necessary for our purposes.

**Figure 3:**
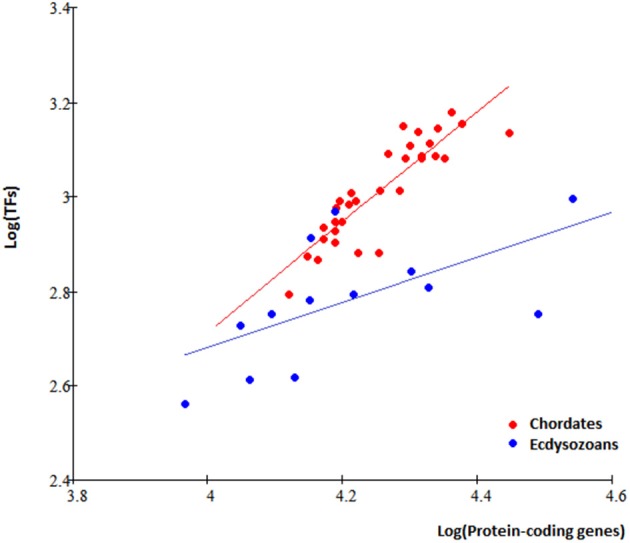
**TFome dynamics in Metazoa: different TFome growth speed in chordates and ecdysozoans**. Chordates: logTF = 1,17logP; Ecdysozoans: logTF = 0.5logP, where P is the number of protein-coding genes and TF is the number of TFs. Each dot represents a species.

In total, 78 TFgFs were detected in metazoan genomes, 58 of them are reliably found in >5 species (Table S7). Expectedly, TFomes of the two groups—chordates and ecdysozoans—are well separated in the plot and have different expansion rates (Figure 3).

**Figure 4:**
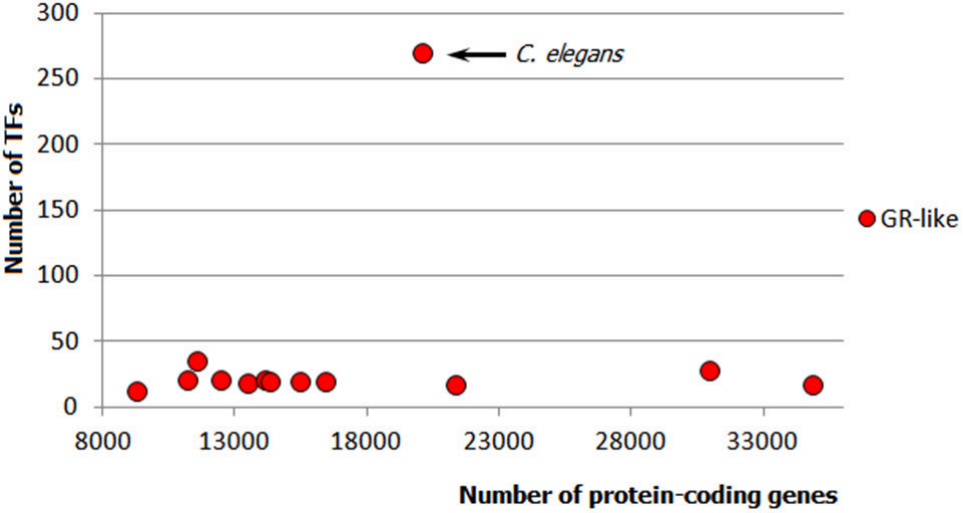
**Example of a single-species expansion**. Glucocorticoid receptor-like TF gene family in animals: only in one species (*C. elegans*) the family expands to 270 TFs, whereas in other ecdysozoan genomes it does not exceed 35. Each dot represents the number of GR-like TFs in one genome.

Apparently, metazoan TFomes can also be subdivided into abundant and small families. Moreover, there is a third category: TFgFs which show a strong expansion in a single genome or just few genomes (in the following, I will refer to them as “single-species expansions”). An example of such single-species expansion is shown in Figure 4: Glucocorticoid receptor-like TFs are represented by about 30 genes in all but one genomes independently of their size; in just one species, however, the family expands to 270 TFs. The single-species expansions do not correlate with the genome size (e.g., in Figure 4, the expansion happened in an average-sized genome). Therefore, they can occur in abundant as well as small TFgF groups. But when averaged over the whole genomes set, the single peaks may give a wrong impression about the abundance of a family. They can also lead to misinterpretation of the family growth tendency if a single but strong expansion occurs in a large genome. So to get a clearer picture by the analysis of the TFgFs growth behavior, it is wise to consider separately the single-peak families from the others when classifying families as abundant.

The single-species expansions are mostly observed in ecdysozoans but are also seen to a lesser extent in chordates and fungi. The largest fungal single-species expansion reaches 27 genes (“C2H2 ZF + HD” TFs) but all other examples are in the range of maximum 5–10 genes (for that reason, we did not subcategorize these families in fungi: they all stayed in the range of small families). In contrast, in arthropods and nematodes, single occasional expansions are quite massive, as was shown in the example of the glucocorticoid (GR) family in *C. elegans* (Figure 4). Thus, single-species expansions can have a significant impact on the whole picture of the TF distributions in ecdysozoa.

**Figure 5:**
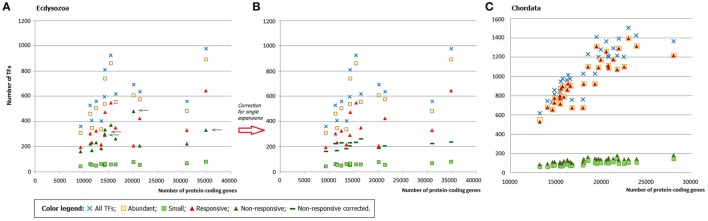
**The role of abundant, small, responsive, and non-responsive families in the TFome dynamics in Metazoa. (A)** Ecdysozoans (Arthropoda and Nematoda). Gray arrows point at the deviating non-responsive families (green triangles), which show unexpected increase. **(B)** Ecdysozoans after the correction for single-species expansions. **(C)** Chrodates.

As in fungi, in ecdysozoans the expansion of the abundant families can account for the whole TFome growth, whereas the input of small families remains practically intact independently of the genome size. However, there is no distinct split of responsive and non-responsive families, up to inverted ratio in several points (Figure 5A). The explanation is in the single expansions: zf-C2H2+GR in *Drosophila melanogaster* and the mosquitos (*Anopheles gambiae* and *Aedes aegypti*), GR, GR+GATA, and DM DNA-binding in *C. elegans*, BESS motif in the aphid (*Acyrthosiphon pisum*) and drosophila. As mentioned above, single-species expansions lead to erroneous conclusions about family abundance and expansion rate. From a statistical point of view, the single-species expansions are outliers so we can introduce a “correction” by simply removing them from the sets (which is a justified operation for outliers). When corrected, the profiles show a better split (Figure 5B). This proves that the single-species expansions were indeed the reason for deviations from the expected distributions. The number of responsive families in arthropods and nematodes is six, which is the same range as in fungi.

In chordates, as we could expect, the picture is qualitatively similar to that in fungi (Figure 5C): we can see a clear split, which illustrates that abundant and/or responsive TFgFs are responsible for the TFome expansion. Quantitatively, however, it differs: the number of abundant (19), as well as responsive families (12, and three further TFgFs appear to be responsive in mammals) is significantly larger than in fungi (Table S8A). But although the number of responsive families in chordates is two to five times higher than in other considered groups, the general principle remains the same: only a limited number of TF gene families expands in response to the genome size growth.

### Using TF signature as a distinguishing taxonomic feature

Each TFome is characterized by a set of TF families and relative portions of each of them (TF distribution). It has long been noticed that TF distributions differ between taxonomic groups (Charoensawan et al., 2010b; de Mendoza et al., 2013; Todd et al., 2014), yet so far this fact has not found a practical application. But the lineage-specific patterns of TFs distribution, TF signatures, are so sensitive to their phylogenetic position that we can use them as distinguishing taxonomic features.

**Figure 6:**
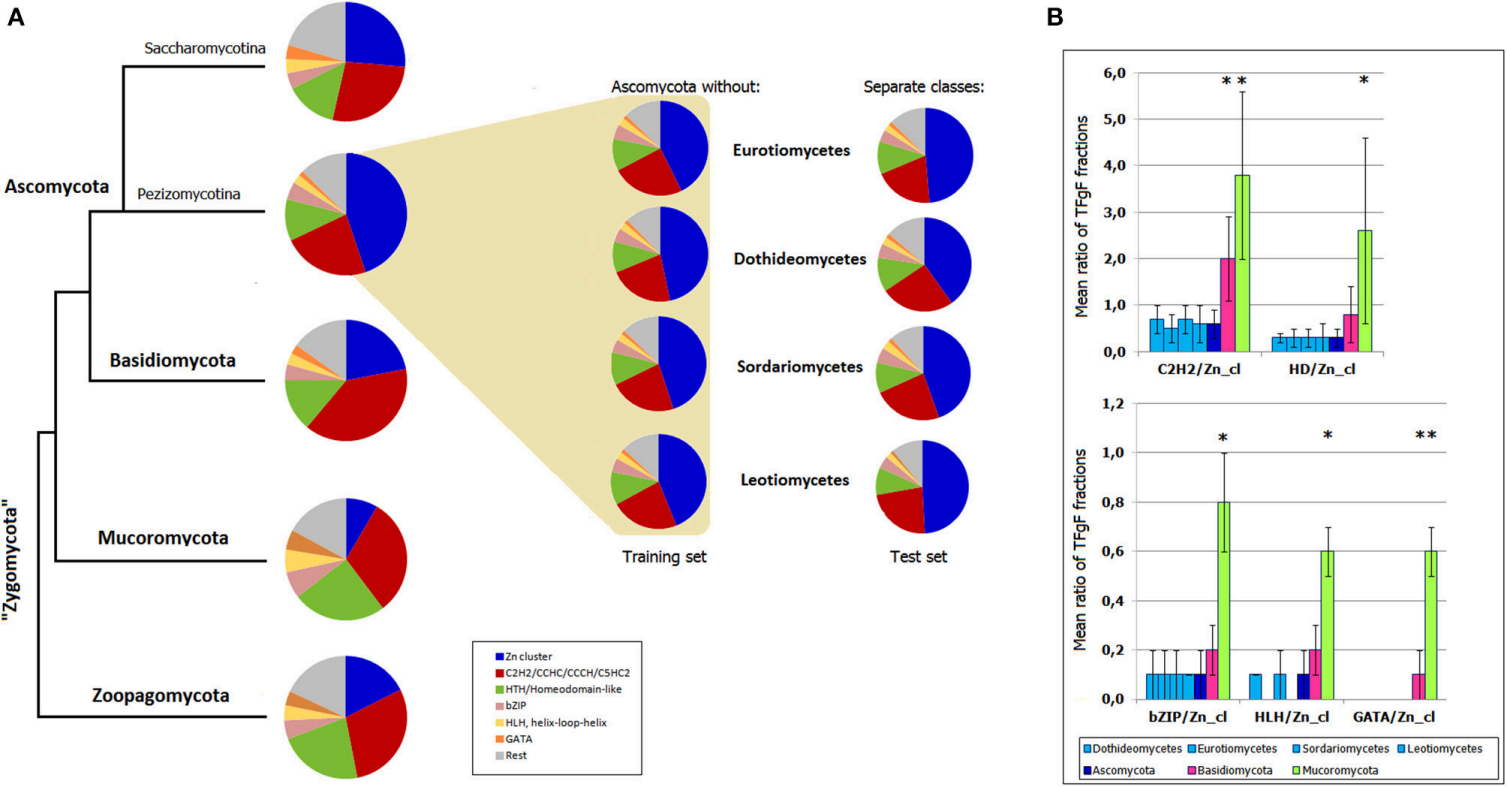
**Predictiveness of TF signatures. (A)** TF signatures based on 6 TFgFs are built for the main fungal phyla and Ascomycota subphyla (Pezizomycotina, of filamentous Ascomycota, and Saccharomycotina). The signatures built for the training sets (Ascomycota without respective class) are shown in the colored field. The test sets (Separate classes) are placed next to the corresponding training sets. **(B)** Statistical significance of the TFgF ratios used for construction of the signatures. Four classes belonging to Pezizomycotina are confronted with three main fungal phyla (Zoopagomycota is not shown because of the small size of the set). Asterisks mark statistically significant differences between the phyla and ascomycete classes.

To see whether TF signatures can serve as models, i.e., have a predictive power, I modeled the situation when a smaller taxon (e.g., a class) is going to be assigned to a one or another phylum. First, TF signatures based on 6 most abundant fungal TFgFs (Zn cluster, C2H2-like, HD-like, bZIP, HLH, and GATA) were constructed for Basidio-, Mucoro-, and Zoopagomycota. Then the same signatures were built for the ascomycete training and test sets. The test sets were four ascomycete classes: Dothideomycetes, Eurotiomycetes, Sordariomycetes, Leotiomycetes. The training sets were obtained by subtraction of the respective class from the whole Ascomycota phylum. In this way, the test data was not used for the model training. The signatures were built for each training and each test set independently and the results are shown in Figure 6A. The signature of the whole Ascomycota phylum is also shown for the fullness of the picture. The signatures of the classes correspond to the signatures of the training sets and differ from the signatures of the other phyla. Slight deviations between the test and training set signatures are too subtle to cause any confusion in assigning the classes to Ascomycota. To show the statistical significance of the signatures, I confronted the ratios of the TFgF fractions that constitute the signatures. The ratios were compared for the four classes, the phylum where they belong (Ascomycota), Basidio- and Mucoromycota (zoopagomycetes had too few representatives to be used for a statistical test; Figure 6B). The TFgF proportions typical for Ascomycota is almost identical in each of the four cognate classes. On the other hand, they significantly differ from the other phyla.

**Figure 7:**
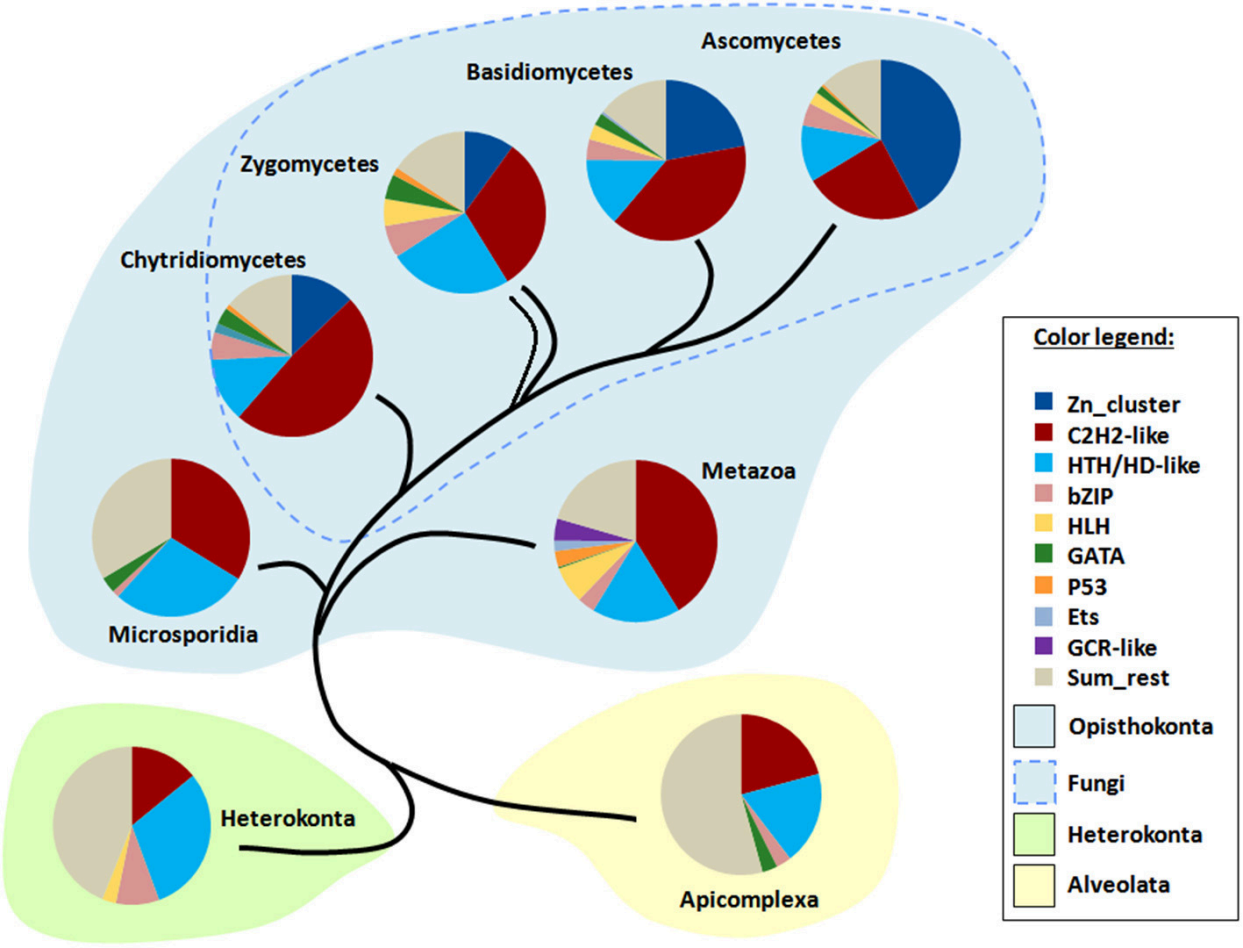
**TF signatures in the phylogenetic background**. 9 TFgFs used for the signature are listed in the color legend.

I applied TF signatures to differentiate the main fungal phyla and also to show the differences to other eukaryotic lineages, including several representatives of Protozoa (Figure 7). The content of a TF signature depends on the phyla to be distinguished; as I was interested in resolving of a large range of genomes from fungi to animals to protists, the signature included several pan-eukaryotic TFs (C2H2, HD, bZip, GATA, HLH), which means that they are found in almost all eukaryotic genomes (de Mendoza et al., 2013) and thus provide a reliable and stable background for comparison. Additionally, fungal- and metazoan-specific TFs such as Zn clusters and GR, respectively, were also taken. In total, 9 TFgFs were used (Figure 7).

The signature provides a clear discrimination between the phylogenetic groups in question: four fungal lineages (Ascomycota, Basidiomycota, Zygomycetes (merged Mucoromycota and Zoopagomycota), Chytridiomycota) as opposed to Microsporidia, Metazoa, Heterokonta, and Apicomplexa (Figure 7). The main distinguishing feature in fungi is the proportion of Zn cluster and two other largest TFgFs: C2H2-like and HD-like. Indeed, the role of Zn cluster dramatically changes from basal to derived lineages: if in chytrids and zygomycetes the fraction of the Zn clusters is much smaller than that of C2H2, in ascomycetes Zn clusters are more numerous than all other TFgFs. In the absence of Zn clusters outside fungi, the interplay of other signature counterparts effectively distinguishes between Metazoa, Microsporidia, and two protist phyla. I should emphasize, however, that because fungi are the main focus of this paper, the signature was mainly adjusted to them; to make a sensitive signature for animals or for protists one should use other TFgFs.

### Dual specificity transcription factors

TFs are characterized by their DNA binding specificity; in a standard TF, there is just one DBD. The existence of TFs with two or more DBDs is documented and some examples have been investigated in details, still such dual-specificity TFs are usually regarded rather as exceptions. To see whether they are really rare sporadic events or a common TFome feature, I analyzed the number and occurrences of TFs with dual DNA-binding specificity (for simplicity, dsTFs) and showed that these TFs are ubiquitous and constitute a small but stable fraction of TFomes (1–4%; Tables S4B, S7B). The analysis reveals 12 dsTF types in fungal genomes and 9 in metazoan, with four types shared between fungi and animals (Table 3). Not surprisingly, the combinations mostly comprise the main TFs that form the main abundant groups and shape the TF repertoires: HDs, Zn fingers, Zn clusters (in fungi), GR (in animals), GATA and bZIP (Table 3). Six of nine metazoan dsTF types are found in the majority (>70%) of the considered genomes, four of them are ubiquitous (present in 96–100% of genomes, Table 3). In fungi, two families are found in 60–70% of all genomes. Although reliably represented, dsTFs are not abundant in genomes, comprising on average 1% and 4% of TF repertoires in fungi and animals, respectively. In animals the portion is higher mostly because of strong single-peak expansions: for instance, zf-C2H2+GR-like, which is in general an insect-specific TF type, is largely expanded in mosquitoes (*A. gambiae* and *A. aegypti*; up to 137 genes). As for standard (non-dual) TFs, single-species expansions of dsTFs can be also observed in fungi but to a lesser extent: e.g., C2H2 ZF+HD reaches 27 genes in *Sclerotinia sclerotiorum*.

**Table 3:** **Dual-specific TFs in fungal and metazoan genomes**.

	**Fungi**		**Animals**		**Comments**
	**Genomes with TF (%)**	**Max**	**Genomes with TF (%)**	**Max**	
Zn cluster+C2H2-like ZF	140 (73)	23	–		Ubiquitous in fungi
C2H2-like ZF+HD	115 (60)	27	53 (96)	7	Ubiquitous
Zn_cluster+C2H2-like+HD	64 (34)	3	–		Ascomycete-specific
GATA+HD	60 (31)	3	6 (11)	2	F: Rare in ascomycetes. M: Not in chordates
Zn_cluster+HD	35 (18)	3	–		
Copper fist+Zn cluster	29 (15)	2	–	–	Not found in zygomycetes
bZIP+HLH	22 (12)	2	–	–	
Zn cluster+bZIP	10 (5)	1	–		
bZIP+C2H2	9 (5)	1	42 (76)	5	F: Not found in basidiomycetes. M: Higher in fish, not in worms.
HD+lambda repressor-like	7 (4)	7	39 (71)	9	F: Mostly found in zygomycetes. M: Not in insects; higher in fish; expansion in lancelet
bZIP+HD-like	5 (3)	1	–	–	Not found in zygomycetes
GATA+Zn cluster	5 (3)	1	–	–	
GR-like+GATA	–	–	55 (100)	13	Ubiquitous in Metazoa
HD+GR-like	–	–	55 (100)	19	Higher in fish
CUT, HD+CUT	–	–	53 (96)	12	Higher in fish
zf-C2H2+GR-like	–	–	12 (22)	137	Insect-specific
C2H2+GATA	–	–	9 (16)	2	Arthropoda-specific

*The analysis was run in 191 fungal and 55 animal TFomes. No dual-specific TFs were found in Microsporidia and protists with one exception of an expansion of GR-like+GATA in amoeba. In fungi, the dsTFs are almost totally absent in yeast (Saccharomycotina as well as Taphrinomycotina) The number in parentheses shows the percentage of genomes in the respective sets (fungal or metazoan), which possess the TF. “Max” is the maximal number of TFs per species. F, fungi; M, metazoa*.

**Figure 8:**
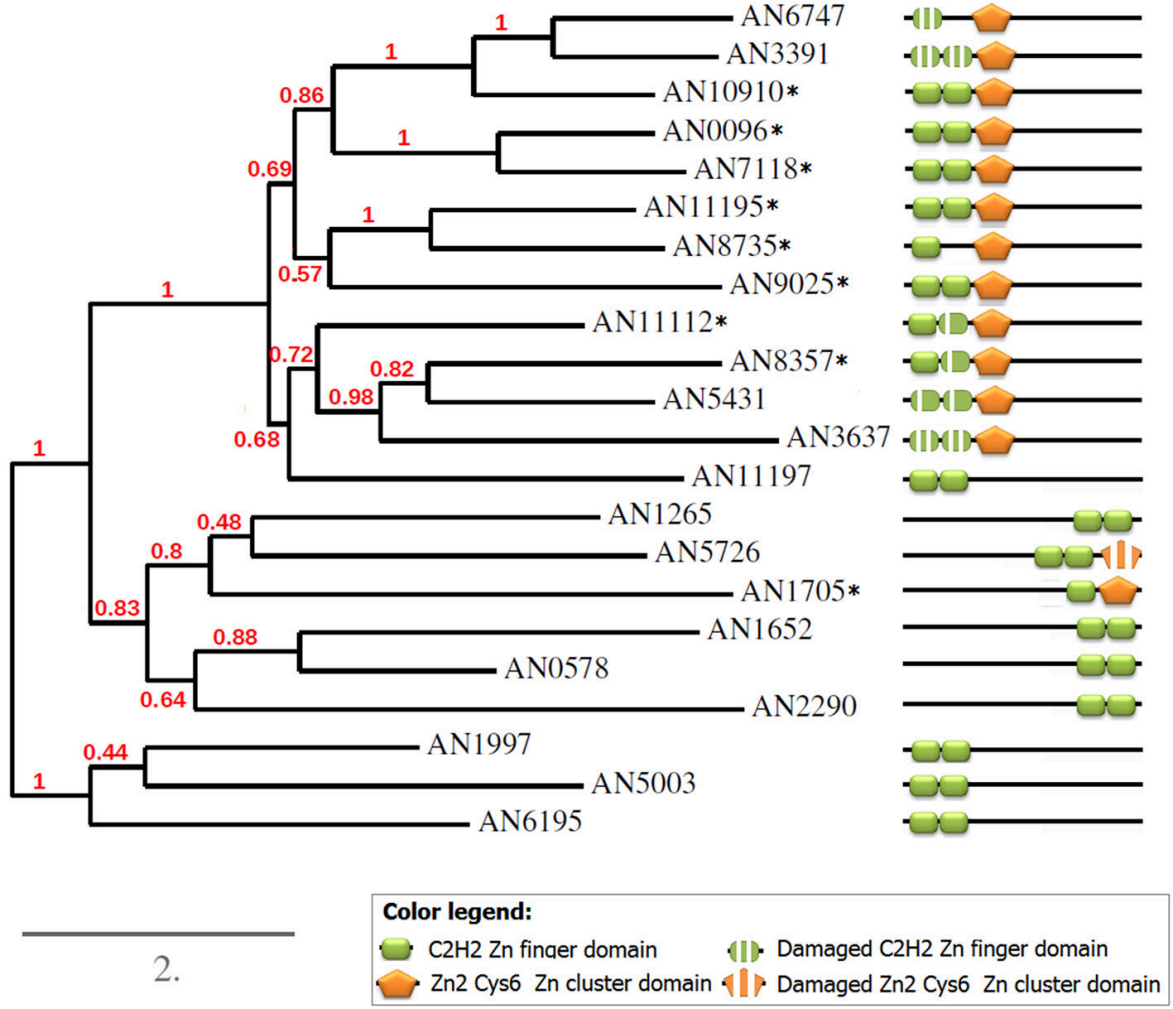
**Phylogeny and domain structures of ***A. nidulans*** C2H2+Zn cluster dual-specificity TFs and their paralogs**. The dual specificity TFs are marked with asterisks.

None of the dsTF families shows steady expansion in response to the genome growth (Tables S5, S8). However, the families are not frozen and show some deviations in the family size (independent of the genome size). It was therefore interesting to look at the phylogenetic relationships of these factors within one genome, in particular to see whether all TFgF representatives are in-paralogs or have different origins. For this analysis, I considered the example of C2H2 ZF+Zn cluster TFs in *Aspergillus nidulans*, where the family expanded to 9 TFs. All 9 proteins have the same domain architecture: two tightly located C2H2 domains are followed by a single Zn cluster. This construction is located N-terminally in all but one dual TFs (Figure 8); all TFs with this conserved domains location were reciprocally identified as paralogs by homology-prediction tool MetaPhOrs (Pryszcz et al., 2011). Thirteen further proteins were suggested by MetaPhOrs as paralogs of dual TF. According to the domain annotation run by InterProScan, they were either Zn cluster or C2H2 ZF factors.

**Figure 9:**
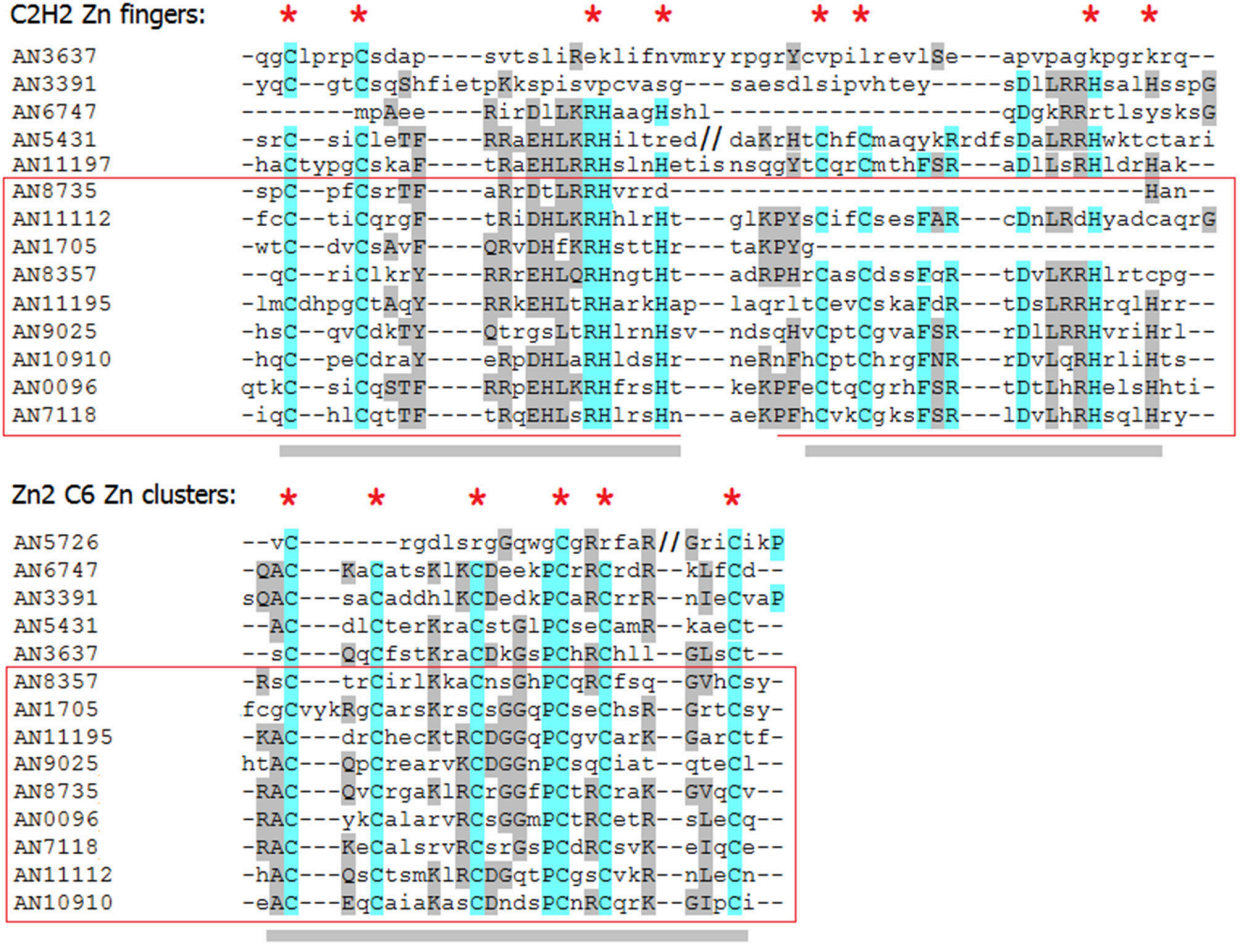
**Alignment of the domain regions of ***A. nidulans*** C2H2+Zn cluster dual-specificity TFs and their paralogs**. Gray bars underline the domains; red asterisks mark the key residues essential for binding. The dual TFs are in the red frame.

To better understand the relationships between all detected paralogs, I reconstructed the phylogeny of this group (Figure 8). As expected, all 8 predicted paralogs get into one clade. But surprisingly, 5 single-DBD TFs also clustered together with the dual TFs. Since the location of the single domains in these TFs was similar to that in the dual TFs, I hypothesized that these proteins could have had the second domains but have lost them in the course of evolution. Indeed, a scrupulous analysis of the sequences around the existing domains revealed remainders of the missing second domains (Figure 9). Interestingly, these are always the C2H2 domains that are missing in this clade. Some of them are still quite well preserved (e.g., in AN5431), however lacking the key residues makes them non-functional.

Aligning the domain structures, existing and lost, with the phylogenetic tree allows us to see the history of the changes (Figure 8). Two TFs with damaged C2H2 domains, AN6747 and AN3391, seem to be the result of a recent duplication of the dual TF AN10910. The second C2H2 is almost completely lost in AN6747 but is still recognizable in AN3391; both proteins have only halves of the first domains (see the alignment in Figure 9). These half-domains do not coincide (AN6747 lacks both cysteins, whereas AN3391 lacks the histidins), which means that the loss happened after the duplication.

In the subclade “AN11112-AN3637,” all proteins have problems with C2H2 domains: in dual TFs, the second C2H2 domains lack the second histidine, which is an essential residue for the binding. Similarly, AN5431, a single-Zn cluster TF, lacks the same second histidines but already in both C2H2 domains. In the more ancient AN3637 the changes affected the C2H2 domains more severely. The history of the C2H2 changes observed in this clade may be a good example of the gradual loss of first the functionality and then the domain itself (we will return to this in Discussion).

The only dual TF with the domains shifted to the C-terminus (AN1705) is found in a different part of the tree, suggesting that it evolved independently of the main group. One of the two proteins that form one clade with AN1705 is most likely also an “ex-dual” TF with some fragments of the Zn cluster near the C2H2 (Figure 9). Other TFs in this clade, as also in the last remaining clade of the tree, are “pure” C2H2 Zn fingers. The alignments gave no hints on any Zn cluster fragments in them.

## Discussion

With ever-growing number of sequenced genomes, regular update of genome-wide annotations is useful to estimate the robustness of our knowledge and to learn new genomic features. This, of course, applies to TFs. The genome set used in this work is nearly two-times larger and with less ascomycete bias than in earlier studies; additionally, the *de novo* TF search was based on a newly collected set of TF-type DBDs (Table S7).

The number of TF families reliably detected in more than 200 fungal genomes (~80 DBD families and about the same amount of TF gene families) is nearly twice as large as that which was found in ~60 genomes 8 years ago (37 families, Shelest, 2008). Interestingly, inspection of new genomes did not uncover any new fungal-specific TFs in addition to those four families that were already known, even though new lineages were added to the search (most of basidio- and zygomycetes were not considered in previous analyses). In general, the sets of TF families in different fungal phyla practically do not differ: each comprises about 80 families and very few of them are phylum-specific. Looking at them from an evolutionary perspective, we can see the obvious traces of history: more basal zygomycetes retain several families that are found also in other eukaryotes but got lost in the rest of fungal lineages, whereas the derived lineage of ascomycetes includes a new specific family, Matα1. The lists of fungal TFs are a useful source of information, especially for evolutionary studies. We can expect interesting insights into the evolution of TFs with the advent of more genomes, especially from protozoan species.

Although the total number of TFs generally responds to the increase of the total number of protein-coding genes, the great majority of TF families are “frozen” (non-responsive), or their expansion is so slow that it cannot provide a significant contribution to the TFome's increment. Apparently, a small set of non-frozen TF families must be responsible for all huge changes in TFome size (up to 30 times). Indeed, we can see that in fungi only five families are not only responsive, but also determinant of the overall growth of TFs number. Moreover, in fact, the overall TFome dynamics can be explained by just three TFgFs: C6 Zn cluster, C2H2 Zn finger, and HD-like. These are also the three largest families in all fungal species. Obviously, if we have an initial non-even distribution of family sizes and allow random independent duplication of any gene from any family, the more abundant families will grow more. However, these considerations cannot explain how the families *become* more abundant (forming that “initial” non-even distribution) and how the newly born families take over their “leading role,” like it happened to Zn clusters. In the initial phases of their emergence (e.g., in chytrids and zygomycetes), the Zn clusters were not abundant at all, so there must have been a particular mechanism of their preference leading to their expansion. I hypothesize that this preference was based not only on the protein or genomic properties but on the properties of the DNA binding sequence (e.g., abundance and recognition effectiveness). In the case of Zn clusters, the basic TF binding site is a pair of three-nucleotide repeats (inverted, everted, or direct repeats, see Marmorstein et al., 1992; Zhang and Guarente, 1994; Hellauer et al., 1996) separated by a number of specifically strictly conserved nucleotides, which largely define the specificity of the binding (Reece and Ptashne, 1993); there are deviations from this basic structure (e.g., NirA binding site, Strauss et al., 1998; PrnA binding site, Gómez et al., 2002) but the linker-separated repeats remain the most widely recognized pattern. This construction allows a highly-specific recognition but is flexible enough to produce a large number of different variants. The discussion of successfulness of different recognition patterns is out of the scope of this paper; I only assume that they could play a certain role in the support of selection toward specific TF types. We will return to the particular role of Zn clusters below.

Thus, in fungi, a handful of abundant families are responsible for most of TFome changes and dynamics. We could assume that fungi are not very different from other eukaryotes in this respect, at least not to the extent of having totally distinct mechanisms of shaping the regulatory machinery. So it was interesting to look how the TFomes grow in other eukaryotic divisions and whether or not they follow the same lines of the TFome shaping. I selected metazoan species, for which the TF annotations were immediately available in the DBD database. The inspection of the new TFomes confirmed that the main idea—that TFomes expand through just a limited number of responsive families—holds true for the Metazoa. But there are also some differences. As expected, in more complex organisms the number of abundant TF gene families is higher and most of them respond to genome size changes. Instead of the 3–5 responsive families that we observed for fungi, 15 families contribute to the TFome expansion in chordate animals. Yet, the overall mechanism of growth in chordates seems to be similar to fungal: the expansions of the responsive families affect more or less all species in the phylum, in a progressive manner with the genome increase, while single-species expansions are not typical. I must admit, however, that the chordate group is very uniform with a majority of mammals and only some fishes, one bird, and one amphibian (the set is limited to the species available in DBD). Possibly, the picture will change with the addition of more genomes from other classes. On the other hand, the ecdysozoa (arthropods and nematodes) are also represented mostly by the insect class, which does not prevent the group from showing a different behavior in terms of TFome growth mechanisms: single-species expansions play a substantial role, so the focus is partly shifted from continuously growing families to those expanding sporadically in one or another species (or genus). These families certainly also contribute to the TFome increase but there is no apparent connection to the proteome size. This difference between chordates and arthropods/nematodes is quite unexpected. The effect of single-species expansions can possibly be attributed to the smallness and relative heterogeneity of the ecdysozoan set; unlike chordates, they contain species of quite distant classes, so a seemingly single expansion may in fact represent a lineage-specific gradual growth, which is not seen as such due to the lack of other representatives. The situation, obviously, can be resolved with more data. All in all, the results show that in Metazoa the picture is more complicated and diverse in comparison to fungi.

The analysis of eukaryotic TFomes suggests that the prevalence of particular families in individual TF profiles is lineage-dependent. This property can be used as a taxonomic feature, and we can construct TF signatures (lineage-specific patterns of TFgF distribution) by selecting those TF families, which are most sensitive to their taxonomic position. The TF signatures can be very informative for taxonomic allocation of a phylum, class or even a species (although the latter may be risky because of large individual deviations). Apparently, not all TFgFs should be included in a TF signature; frozen and weakly represented families with a patchy distribution within a lineage are not relevant in this context. The number and quality of TFgFs serving as a TF signature should depend on the set of taxa to be described or distinguished. The three main fungal families (HD, C2H2-like, and Zn cluster) can discriminate the main fungal lineages and help with allocation (or not) of some questionable phyla to the fungal kingdom (Figure S2). However, to resolve the differences between non-fungal species that lack Zn clusters, just three families may be insufficient. In this work, I used 9 TFgFs to build a “broad” signature to differentiate a very diverse collection of phyla, from fungi to protists. This broad signature included three fungal and metazoa-specific TFgFs, otherwise it comprised ubiquitous families to grant a common basis for the comparison. The 9-TFs signature provides a clear separation for the selected phyla; additionally, it allows us to make some observations of similarities and differences of the analyzed taxa. The TF signatures of all considered eukaryotic groups are dominated by the same set of the largest TF families: C2H2-like, Homeodomain-like, and - in fungi - Zn cluster. Small and medium-size families that rise in one lineages and vanish in the other (e.g., Ets and GR are specific to animals, etc.) also play their discriminating role but the major dramatic changes concern the “main” families. We can see how the roles of the main TFgFs have dynamically altered through evolution. HD and C2H2 are the most numerous in all eukaryotes—at least we can see it in all so far considered examples. In fungi, a novelty, Zn clusters, appeared and started to steadily take over the superior (in numerical sense) role. The TF signatures clearly reflect this process: the fraction of Zn clusters grows from very small (~10%) in chytrids and zygomycetes, to modest in basidiomycetes (~20%) and to dominating in younger lineages of Ascomycota (Pezizomycotina) (~40%). (Of note, Zn clusters do not prevail in Saccharomycotina and Taphrinomycotina). We can assume that Zn clusters reached preeminence via rewiring existing regulatory circuits because they regulate most of the metabolic processes (both primary and secondary metabolism), which are present in all cells and are thus unavoidably regulated in earlier, Zn cluster-free lineages, apparently by other TFs. As shown above (section How does the number of TFs grow?, Figure 2B), the TFomes of filamentous ascomycetes grow practically on account of Zn clusters, which suggests that they also take over regulation of new functions that arise with the variety of life styles.

The TF signatures should be most useful for fine tuning the assignment of phyla with unclear taxonomic position. This is particularly useful for fungi with their complex and not finally resolved taxonomy. There are some taxonomic groups, the assignment of which to fungi is still under debate; Microsporidia are an exemplarily case, which shows the effectiveness of TF signatures. The TF signature (Figure 7) clearly suggests that Microsporidia are not a fungal phylum: the distribution pattern is far from typically fungal, starting with the complete absence of Zn cluster TFs. An additional indication of non-fungal nature of Microsporidia is that their TF repertoire is very poor in comparison to fungal, as they lack almost half of families found in fungi. Not going into further discussion of Microsporidia allocation, I just want to demonstrate that TF signatures can provide additional evidence in this kind of debate.

The TF signatures reflect the ratios of the means (or medians). Of course, each particular species may deviate, sometimes significantly. For this reason, the approach is not applicable for single species assignment.

Among all described TF gene families one is particularly intriguing. These are dual-specificity TFs, transcription factors with two or more DNA-binding domains of different types. Principally, the idea is not totally new: as I mentioned in Introduction, some DBD combinations have been already well characterized. However, some basic questions remain unanswered, e.g., how typical are these TFs for eukaryotic genomes; how they are distributed; which domains constitute dual TFs? Not aiming at a comprehensive investigation of the dual TFs in this work, I tried to answer these and some further questions.

The present study shows that the dual TFs are not a rare event, although they seem to be confined to Metazoa and fungi: all metazoan and almost all fungal genomes contain these factors. Most of the dual TFs show lineage specificity (Table 3), which cannot be explained by the availability of the constituent domains. The list of the involved domains is quite limited but they are not restricted to particular phyla where the respective dsTFs occur (Table 3). Of 10 included DBDs, seven are ubiquitous in Eukaryotes and three (Zn cluster, copper fist, and GR-like) are specific for either fungi (the two former) or animals (GR-like). Dual TFs are totally absent in microsporidia and they are also exceptionally rare in the Saccharomycotina and Taphrinomycotina. The latter two lineages have the same repertoire of TF families as other ascomycetes but the dual TFs do not occur. Small genome size, primitive life style (especially for parasitic species) and generally low number of TFs could be an explanation.

Dual TF gene families do not correlate with the genome size, although their cognate single-DBD TFgFs belong to the abundant and responsive groups. Hence, the expansion strategies of single counterparts do not influence the abundance of the dual combinations. This observation suggests that dual TFs are mostly not formed *de novo* by random fusions (in which case the probability of the dsTF occurrences would depend on the frequency of the single counterparts and would be predictable) but evolve by duplications same as single-DBD TFs. To get a better idea of the relationship between dsTFs paralogs, I analyzed in more detail one family of dsTFs in one species, taking as an example C2H2+Zn cluster TFs in *A. nidulans* (C2H2+Zn cluster is the most frequent dsTFs type in fungi; Figures 8, 9). This analysis revealed an interesting history of gene duplications accompanied by multiple independent domain losses. The phylogenetic tree reconstructed for the 22 predicted paralogs (including dual- as well as single-DBD TFs) suggests that 8 of 9 dsTFs of this species have indeed evolved from the same origin by duplications; they form one clade in the tree and share the same domain architecture. Moreover, the group of these TFs has been larger: several TFs of the same clade, which apparently have also evolved by duplication of the dsTFs but possess only one DBD now, have had the second domains but lost them recently. The remnants of the domains, partly well conserved, can still be found in their sequences (Figure 9). In fact, we observe the process of shrinking of the dsTF family, with some members changing their specificity and possibly the function.

In one of the clades (AN11112-AN11197) the process of the domain loss can be seen “by stages.” The gene at the base of the branch, AN11197, lacks the Zn cluster domain and has no traces of it. The structure of AN11197 gives no hints on whether the Zn cluster has been lost or it never has been there and was fused in later genes (starting with AN11112). However, AN111197 is identified by MetaPhOrs as a paralog of several dsTFs and is insistently clustered with dual TFs by aligning and tree-building programs; this suggests that the first scenario (total loss of the Zn cluster) is more probable. This speculation needs, of course, more support and comparative analysis with other Aspergillus species could be helpful for understanding these details. Apart from AN11197, all other genes in the clades are dual (or “ex-dual”) TFs with C2H2 domains damaged to different degree. Possibly, the second histidine of one of the C2H2 domains has been lost before the duplication events: it was already missing in the AN11112, which is in the base of the branch. The most recent gene, AN8357, inherited exactly the same domain structure. This stability suggests that the functionality of the DBD combination has not been affected. However, the other in-paralogs lost further key residues after the duplications: AN5431 got an insertion that probably substituted or shifted the H in the first C2H2 domain, whereas AN3637, which is the result of an earlier duplication and had more time for changes, has got massive losses in both C2H2 domains. Most likely, we observe here the active process of neofunctionalization of the paralogs.

Independent domain losses occurred also in the other parts of the tree (clades AN6747-AN10910 and AN1265-AN1705). What is the real meaning of these changes and which influence they have on the regulatory function of the TFs, should be answered by further investigations.

Dual specificity TFs are interesting from both evolutionary and functional points of view. One of the most important questions, which has to answered for each dual TF type, is whether both DBDs still bind to DNA and if yes, if this is simultaneous or alternative binding. In the considered example of the C2H2+Zn cluster TFs, all three DBDs are located on the N-terminus in close proximity to each other. Such location is typical for Zn fingers; in general, C2H2 domains tend to occur in repeats separated by a short sequence. They also have a known tendency to cooperate with similar Zn fingers, which in this case might have been substituted by a Zn cluster. We can assume that these three domains may cooperate for DNA binding. An involvement of both C2H2 and Zn cluster in regulation, but not their synergetic cooperation, has been shown for a similar construction in Cmr1p TF (Tsuji et al., 2000). But in the absence of direct experiments the details of the DNA recognition by this particular domain combination remain obscure. On the other hand, intensive domain losses may be a sign of neofunctionalization of the proteins; further experiments are needed to show whether all paralogs are functional and whether they retain the transcription regulation activity, and in which processes they are involved.

In conclusion, this paper updates and summarizes our present knowledge about composition and expansion strategies of fungal TFomes. I show for the first time that in fungi as well as in animals only a small set of TF gene families defines the TFome expansions. The lineage specific TFgF distribution is shown to be a useful taxonomic feature sensitive to inter-phyla differences. Finally, I demonstrate that an earlier under-estimated class of TFs with dual DNA binding specificity is in fact ubiquitous in fungi and metazoa, well represented in different lineages and has an interesting evolutionary history. A detailed analysis of just one representative family of dual-specificity TFs reveals an intriguing story of domain losses and possibly neofunctionalization of paralogs.

## Materials and methods

### Data sources

Fungal data: InterProScan annotations were downloaded for all published fully sequenced genomes from Mycocosm portal of JGI (http://genome.jgi.doe.gov/programs/fungi/index.jsf, Grigoriev et al., 2014). The list of used genomes and corresponding references can be found in Table S1.

Proto- and Metazoan data: TF tables were downloaded for a manually-selected non-redundant set of species from DBD database (DBD DB; http://www.transcriptionfactor.org, Wilson et al., 2008; Table S2). For TF signature analysis, Apicomplexa and Heterokonts (oomycetes and diatoms) species were taken as protozoan representatives.

### TF predictions

TF predictions and annotation of the corresponding DNA-binding domains (DBD) were made by confronting a collection of TF-type DNA-binding domains with genome-wide protein domain prediction tables downloaded from JGI and DBD DB. The tables contain the domain information for each protein of a genome.

DNA-binding domains can be divided into two groups, those that occur in TFs (TF-type DBDs) and that occur in other DNA-binding proteins. In this work, we are interested only in the former. The manually curated collection of TF-type DBD (Table S9) was initially based on the domain set from DBD DB but then updated using InterPro; the list was manually cleaned from all non-TF DNA-binding domains.

The genome-wide domain prediction tables from JGI and DBD DB were searched for coincidences with the TF-type domain collection using GNU R (https://www.r-project.org/) scripts. A protein was considered as a TF if it had at least one TF-type DNA-binding domain. The procedure was run separately for JGI and DBD DB datasets.

TF genes were merged in groups around “dominant” DBD, for instance all genes with HD-like DBDs were put into the HD group. The dominant DBD is the one that is the most abundant within the group; for simplicity, we retain the name “TF family” for such groups.

Dual-specificity TFs were defined as following:

- TFs with two or more DBDs belonging to different TF classes; the exception was made for combinations of HD and CUT and HD + lambda-repressor like (which belong to the same HTH class), because these are known dual-specificity TFs. Note that C2H2, CCHC, and CCCH Zn fingers were merged in one group in this analysis.
- TFs must be represented in ≥5 genomes.
- Consequently, a candidate dsTF was merged with an existing group if it was represented by non-significant number of genes on the background of the corresponding single-DBD TFs; for instance, a combination “Zinc finger, GATA-type + bZIP” was represented by just one gene on the background of ~5,600 bZIP TF and ~3,000 GATA TF genes found in all fungal genomes (in total), so this gene was merged with the bZIP group.
- If there was no group to merge, the insignificantly represented dsTFs could be retained but did not influence the analysis because of their negligible input.

### TFome dynamics analysis

The responsiveness of TFgFs to the proteome size growth was characterized by the exponent of the power law of TFgF distribution for each family, with the threshold of *exp* = 0.5 for being considered as growing. With exponents lower than that, the growth is so slow that can be neglected. This analysis was followed by manual inspection to eliminate misinterpretations (such as in cases of single-species expansions). The coefficient of determination was calculated by the formula:

$$\begin{array}{l} R^{2}=1-\frac{\sum {\left(y_{i}-f_{i}\right)}^{2}}{\sum {\left(y_{i}-ȳ\right)}^{2}} \end{array}$$

where *y*_*i*_ is the number of TFs in the *i*-th genome, ȳ is the mean of the observed data, and *f*_*i*_ is a correspondent modeled value.

The threshold for fungi was taken as 0.5 (standard threshold).

The analysis was run for all families that fulfilled the following conditions: (i) the family must be present in >10 species; (ii) the maximal number of TFs per genome must exceed 5 in at least one genome. The latter condition eliminates the cases of small increments like from 1 to 4 genes, which are of no interest for this study. Genomes with no representatives of the considered family were ignored by the exponent calculation (so only meaningful genomes were taken into account for each family).

### Inspection of paralogy of selected dsTFs

Each dsTF protein of interest was submitted to MetaPhOrs (http://orthology.phylomedb.org/, Pryszcz et al., 2011) paralog search in *A. nidulans* genome. In parallel, proteins were submitted to Phylome DB Blast to search for corresponding phylomes (http://phylomedb.org/, Huerta-Cepas et al., 2014).

### Alignments and phylogeny

The sequences were aligned with Muscle (Edgar, 2004), the ML tree was constructed by PhyML v3.0.1 (Guindon et al., 2010), with statistical branch supports computed with aBayes likelihood-based method.

## Author contributions

ES is the sole author of this paper. She designed the study, collected data, ran all calculations, analyzed the results, wrote the paper and approved it for publication.

## Funding

This work was supported by Collaborative research centers ChemBioSys (CRC 1127 ChemBioSys) and CRC-Transregio FungiNet by Deutsche Forschungsgemeinshaft (DFG).

### Conflict of interest statement

The author declares that the research was conducted in the absence of any commercial or financial relationships that could be construed as a potential conflict of interest.

## Supplementary material

Figure S1 **TF-type DBD families in fungal phyla**. The Venn diagram is made for 80 typical fungal TFDFs.

Figure S2 **Three-family TF signatures are sufficient to distinguish the main fungal lineages**. Microsporidia show a distinct pattern, completely lacking the Zn clusters and having different a proportion of C2H2 and Homeodomains.

Table S1 **List of fungal and microsporidian genomes from JGI**.

Table S2 **Major taxonomic assignments of the TFDFs found in fungi and Microsporidia. (A)** Rare TFFs (found in 1-5 species); **(B)** Typically found in fungi.

Table S3 **List of TFDFs detected in 205 fungal and microsporidial genomes**. Only the families detected in more than 5 species are shown. For abbreviations, see Table S1.

Table S4 **List of TFgFs detected in 205 fungal and microsporidial genomes**.

Table S5 **Responsiveness of fungal TFgFs to the proteome size growth**.

Table S6 **List of proto- and metazoan genomes from DBD**.

Table S7 **TFgFs in metazoa**.

Table S8 **Responsiveness of metazoan TFgFs to the proteome size growth**.

Table S9 **List of DNA-binding domains used for the definition of TFs**.

## Abbreviations

DBD: DNA-binding domain
DB: database
dsTF: dual-specific transcription factor
GR: glucocorticoid receptor
HD: homeodomain
HGT: horizontal gene transfer
HLH: helix-loop-helix
HTH: helix-turn-helix
LECA: least eukaryotic common ancestor
TFDF: transcription factor DBD family
TFgF: transcription factor gene family
WGD: whole genome duplication
ZF: zinc finger.
