# Supplementary material for: Transcription Factors in Fungi: TFome Dynamics, Three Major Families, and Dual-Specificity TFs
Source: Front Genet. 2017 May 4;8:53. doi: 10.3389/fgene.2017.00053 (PMC5415576; doi:10.3389/fgene.2017.00053)
Supplement: Figure S1 — TF-type DBD families in fungal phyla. The Venn diagram is made for 80 typical fungal TFDFs. [file Image1.PDF]

frontiers

Supplementary Material

Article Title Transcription factors in fungi: TFome dynamics, three major families, and dual-specificity TFs

Ekaterina Shelest*

*** Correspondence:** ekaterina.shelest@leibniz-hki.de

Supplementary figure S1.

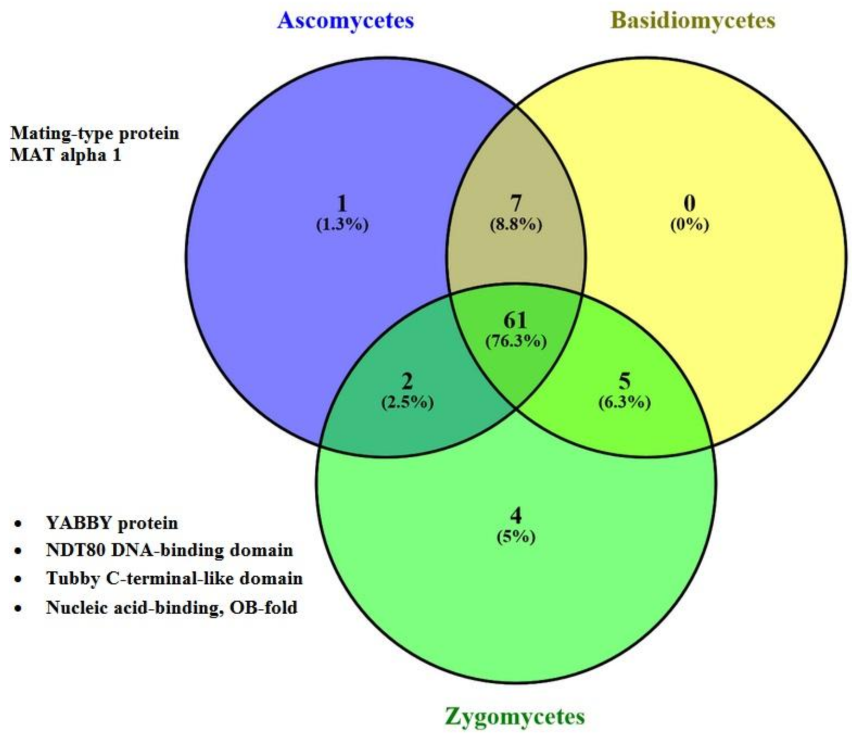

Fig. S1. TF-type DBD families in fungal phyla. The Venn diagram is made for 80 typical fungal DBDFs. Phylum-specific TFDFs are listed at the side of the respective phylum.
