# Supplementary material for: Transcription Factors in Fungi: TFome Dynamics, Three Major Families, and Dual-Specificity TFs
Source: Front Genet. 2017 May 4;8:53. doi: 10.3389/fgene.2017.00053 (PMC5415576; doi:10.3389/fgene.2017.00053)
Supplement: Figure S2 — Three-family TF signatures are sufficient to distinguish the main fungal lineages. Microsporidia show a distinct pattern, completely lacking the Zn clusters and having different a proportion of C2H2 and Homeodomains. [file Image2.PDF]

Supplementary Material

Article Title Transcription factors in fungi: TFome dynamics, three major families, and dual-specificity TFs

Ekaterina Shelest*

*** Correspondence:** ekaterina.shelest@leibniz-hki.de

Supplementary figure S2.

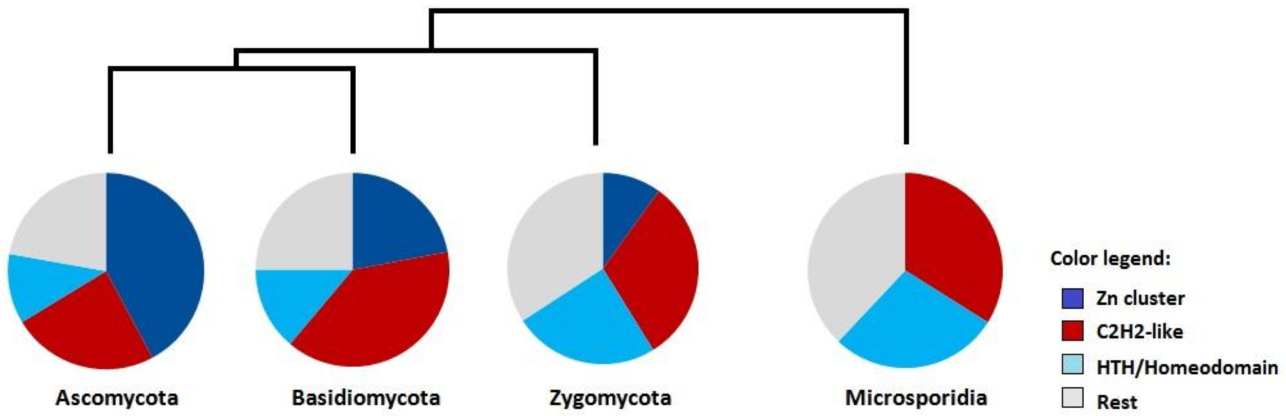

Fig. S2. 3-family TF signatures are sufficient to distinguish the main fungal lineages. Microsporidia show a distinct pattern, completely lacking the Zn clusters and having different a proportion of C2H2 and Homeodomains.
