# Supplementary material for: Transcription Factors in Fungi: TFome Dynamics, Three Major Families, and Dual-Specificity TFs
Source: Front Genet. 2017 May 4;8:53. doi: 10.3389/fgene.2017.00053 (PMC5415576; doi:10.3389/fgene.2017.00053)
Supplement: Table S1 — List of fungal and microsporidian genomes from JGI. [file Table1.PDF]

*Supplementary Material*

# Article Title Transcription factors in fungi: TFome dynamics, three major families, and dual-specificity TFs

**Ekaterina Shelest***

*** Correspondence:** ekaterina.shelest@leibniz-hki.de

## 1 Genomic data used in the study

**Table S1**. List of fungal and microsporidian genomes from JGI (http://genome.jgi.doe.gov/programs/fungi/index.jsf ).

| Species name | JGI abbreviation | Reference |
|---|---|---|
| **Ascomycota** | | |
| Arthrobotrys oligospora ATCC 24927 | Artol | Yang J et al., 2011 |
| Arthroderma benhamiae CBS 112371 | Artbe | Burmester A et al., 2011 |
| Ascocoryne sarcoides NRRL50072 | Ascsa | Gianoulis TA et al., 2012 |
| Ascoidea rubescens NRRL Y17699 v1.0 | Ascru | Riley R et al., 2016 |
| Ashbya gossypii ATCC 10895 | Ashgo | Gattiker A et al., 2007; Dietrich et al., 2004 |
| Aspergillus acidus v1.0 | Aspfo | de Vries R |
| Aspergillus brasiliensis v1.0 | Aspbr | de Vries R |
| Aspergillus clavatus NRRL 1 from AspGD | Aspcl | Arnaud MB et al., 2012 |
| Aspergillus flavus NRRL3357 | Aspfl | Arnaud MB et al., 2012 |
| Aspergillus fumigatus A1163 | Aspfu | Fedorova ND et al., 2008, Nierman et al., 2005 |
| Aspergillus glaucus v1.0 | Aspgl | de Vries |
| Aspergillus kawachii IFO 4308 | Aspka | Futagami T et al., 2011 |
| Aspergillus nidulans from AspGD | Aspnid | Arnaud MB et al., 2012 |
| Aspergillus niger CBS 513.88 | Aspni | Pel HJ et al., 2007 |
| Aspergillus oryzae RIB40 | Aspor | Arnaud MB et al., 2012 |
| Aspergillus sydowii v1.0 | Aspsy | de Vries R |
| Aspergillus terreus NIH 2624 | Aspte | Arnaud MB et al., 2012 |
| Aspergillus tubingensis v1.0 | Asptu | de Vries R |
| Aspergillus versicolor v1.0 | Aspve | de Vries R |
| Aspergillus wentii v1.0 | Aspwe | de Vries R |
| Aspergillus zonatus v1.0 | Aspzo | de Vries R |
| Aureobasidium pullulans var. melanogenum CBS 110374 | Aurme | Gostincar C et al., 2014 |

| | | |
|---|---|---|
| Aureobasidium pullulans var. namibiae CBS 147.97 | Aurna | Gostincar C et al., 2014 |
| Aureobasidium pullulans var. pullulans EXF-150 | Aurpu | Gostincar C et al., 2014 |
| Aureobasidium pullulans var. subglaciale EXF-2481 | Aursu | Gostincar C et al., 2014 |
| Babjeviella inositovora NRRL Y-12698 v1.0 | Babin | Riley R et al., 2016 |
| Baudoinia compniacensis UAMH 10762 (4089826) v1.0 | Bauco | Ohm RA et al., 2012 |
| Beauveria bassiana ARSEF 2860 | Beaba | Xiao G et al., 2012 |
| Blumeria graminis f.sp. hordei DH14 | Blugr | Spanu PD et al., 2010 |
| Botryosphaeria dothidea | Botdo | Z. Liu et al., 2016, Marsberg A et al., 2016 |
| Botrytis cinerea v1.0 | Botci | Staats M et al., 2012; Amselem et al., 2011 |
| Candida arabinofermentans NRRL YB-2248 v1.0 | Canar | Riley R et al., 2016 |
| Candida caseinolytica Y-17796 v1.0 | Canca | Riley R et al., 2016 |
| Candida tanzawaensis NRRL Y-17324 v1.0 | Canta | Riley R et al., 2016 |
| Candida tenuis NRRL Y-1498 v1.0 | Cante | Wohlbach DJ et al., 2011 |
| Cenococcum geophilum 1.58 v2.0 | Cenge | Peter M et al., 2016 |
| Cladosporium fulvum v1.0 | Clafu | de Wit PJ et al., 2012, Ohm et al., 2012 |
| Coccidioides immitis RS | Cocci | Sharpton TJ et al., 2009 |
| Cochliobolus carbonum 26-R-13 v1.0 | Cocca | Condon BJ et al., 2013 |
| Cochliobolus heterostrophus C4 v1.0 | Coche | Ohm RA et al., 2012, Condon BJ et al., 2013 |
| Colletotrichum graminicola M1.001 | Colgr | O'Connell RJ et al., 2012 |
| Cordyceps militaris CM01 | Cormi | Zheng P et al., 2011 |
| Cyberlindnera jadinii NRRL Y-1542 v1.0 | Cybja | Riley R et al., 2016 |
| Debaryomyces hansenii | Debha | Sacerdot C et al., 2008 |
| Dekkera bruxellensis CBS 2499 v2.0 | Dekbr | Piskur J et al., 2012 |
| Dothistroma septosporum NZE10 v1.0 | Dotse | de Wit PJ et al., 2012, Ohm et al., 2012 |
| Eurotium rubrum v1.0 | Eurru | Kis-Papo T et al., 2014 |
| Eutypa lata UCREL1 | Eutla | Blanco-Ulate B et al., 2013 |
| Fusarium fujikuroi IMI 58289 | Fusfu | Wiemann P et al., 2013 |
| Fusarium graminearum v1.0 | Fusgr | Cuomo CA et al., 2007 |
| Fusarium oxysporum v1.0 | Fusox | Ma LJ et al., 2010 |
| Fusarium verticillioides 7600 | Fusve | Ma LJ et al., 2010 |
| Glarea lozoyensis ATCC 20868 | Glalo | Chen L et al., 2013 |
| Grosmannia clavigera kw1407 | Grocl | DiGuistini S et al., 2011 |
| Gymnopus luxurians v1.0 | Gymlu | Kohler A et al., 2015 |
| Hyphopichia burtonii NRRL Y-1933 v1.0 | Hypbu | Riley R et al., 2016 |
| Hysterium pulicare | Hyspu | Ohm RA et al., 2012 |
| Kluyveromyces lactis | Klula | Dujon B et al., 2004 |
| Leptosphaeria maculans | Lepma | Rouxel T et al., 2011 |
| Lipomyces starkeyi NRRL Y-11557 v1.0 | Lipst | Riley R et al., 2016 |
| Macrophomina phaseolina MS6 | Macph | Islam MS et al., 2012 |
| Magnaporthe grisea v1.0 | Maggr | Dean RA et al., 2005 |
| Metarhizium acridum CQMa 102 | Metac | Gao Q et al., 2011 |
| Metarhizium robertsii ARSEF 23 | Metan | Gao Q et al., 2011 |

| | | |
|---|---|---|
| Metschnikowia bicuspidata NRRL YB-4993 v1.0 | Metbi | Riley R et al., 2016 |
| Monacrosporium haptotylum CBS 200.50 | Monha | Meerupati T et al., 2013 |
| Myceliophthora thermophila (Sporotrichum thermophile) v2.0 | Spoth | Berka RM et al., 2011 |
| Nadsonia fulvescens var. elongata DSM 6958 v1.0 | Nadfu | Riley R et al., 2016 |
| Neofusicoccum parvum UCRNP2 | Neopa | Blanco-Ulate B et al., 2013 |
| Neosartorya fischeri NRRL 181 | Neofi | Arnaud MB et al., 2012 |
| Neurospora crassa OR74A v2.0 | Neucr | Galagan JE et al., 2003 |
| Oidiodendron maius Zn v1.0 | Oidma | Kohler A et al., 2015 |
| Ophiostoma piceae UAMH 11346 | Ophpc | Haridas S et al., 2013 |
| Pachysolen tannophilus NRRL Y-2460 v1.2 | Pacta | Riley R et al., 2016 |
| Paracoccidioides brasiliensis Pb03 | Parbr | Desjardins CA et al., 2011 |
| Penicillium chrysogenum Wisconsin 54-1255 | Pench | van den Berg MA et al., 2008 |
| Penicillium digitatum PHI26 | Pendi | Marcet-Houben M et al., 2012 |
| Penicillium oxalicum 114-2 | Penox | Liu G et al., 2013 |
| Phaeoacremonium aleophilum UCRPA7 | Phaal | Blanco-Ulate B et al., 2013 |
| Pneumocystis jirovecii | pneji | Cisse OH et al., 2012 |
| Podospora anserina S mat+ | Podan | Espagne E et al., 2008 |
| Pyrenophora teres f. teres | Pyrte | Ellwood SR et al., 2010 |
| Pyronema confluens CBS100304 | Pyrco | Traeger S et al., 2013 |
| Rhytidhysteron rufulum | Rhyru | Ohm RA et al., 2012 |
| Saccharomyces cerevisiae S288C | Scer | Goffeau A et al., 1996 |
| Schizosaccharomyces japonicus yFS275 | Schja | Rhind N et al., 2011 |
| Schizosaccharomyces octosporus yFS286 | Schoc | Rhind N et al., 2011 |
| Schizosaccharomyces pombe | Schpo | Wood V et al., 2002 |
| Sclerotinia sclerotiorum v1.0 | Sclsc | Amselem J et al., 2011 |
| Septoria musiva SO2202 v1.0 | Sepmu | Dhillon B, 2015; Ohm RA et al., 2012 |
| Setosphaeria turcica Et28A v1.0 | Settu | Ohm RA et al., 2012 |
| Spathaspora passalidarum NRRL Y-27907 v2.0 | Spapa | Wohlbach DJ et al., 2011 |
| Stagonospora nodorum SN15 v2.0 | Stano | Hane JK et al., 2007 |
| Talaromyces marneffei ATCC 18224 | Talma | Nierman WC, et al., 2015 |
| Talaromyces stipitatus ATCC 10500 | Talst | Nierman WC, et al., 2015 |
| Taphrina deformans | Tapde | Cisse OH et al., 2012 |
| Trichoderma atroviride v2.0 | Triat | Kubicek CP et al., 2011 |
| Trichoderma reesei v2.0 | Trire | Martinez D et al., 2008 |
| Trichoderma virens Gv29-8 v2.0 | Trivi | Kubicek CP et al., 2011 |
| Trichophyton rubrum CBS 118892 | Triru | Martinez DA et al., 2012 |
| Trichophyton verrucosum HKI 0517 | Trive | Burmester A et al., 2011 |
| Tuber melanosporum from Genoscope | Tubme | Martin F et al., 2010 |
| Uncinocarpus reesii 1704 | Uncre | Sharpton TJ et al., 2009 |
| Verticillium alfalfae VaMs.102 | Veral | Klosterman SJ et al., 2011 |
| Verticillium dahliae v1.0 | Verda | Klosterman SJ et al., 2011 |
| Wickerhamomyces anomalus NRRL Y-366-8 v1.0 | Wican | Riley R et al., 2016 |
| Yarrowia lipolytica (strain CLIB122) | Yarli | Dujon B et al., 2004 |

| | | |
|---|---|---|
| **Basidiomycota** | | |
| Amanita muscaria Koide v1.0 | Amamu | Kohler A et al., 2015 |
| Armillaria mellea | Armme | Collins C et al., 2013 |
| Auricularia subglabra v2.0 | Aurde | Floudas D et al., 2012 |
| Bjerkandera adusta v1.0 | Bjead | Binder M et al., 2013 |
| Botryobasidium botryosum v1.0 | Botbo | Riley R et al., 2014 |
| Calocera cornea v1.0 | Calco | Nagy LG et al., 2016 |
| Calocera viscosa v1.0 | Calvi | Nagy LG et al., 2016 |
| Ceriporiopsis (Gelatoporia) subvermispora B | Cersu | Fernandez-Fueyo E et al., 2012 |
| Chaetomium globosum v1.0 | Chagl | Berka RM et al., 2011 |
| Coniophora puteana v1.0 | Conpu | Floudas D et al., 2012 |
| Coprinopsis cinerea | Copci | Stajich JE et al., 2010 |
| Cryptococcus neoformans var neoformans JEC21 | Cryne | Loftus BJ et al., 2005 |
| Cylindrobasidium torrendii v1.0 | Cylto | Floudas D et al 2015 |
| Dacryopinax sp. DJM 731 SSP1 v1.0 | Dacsp | Floudas D et al., 2012 |
| Daedalea quercina v1.0 | Daequ | Nagy LG et al., 2016 |
| Dichomitus squalens v1.0 | Dicsq | Floudas D et al., 2012 |
| Exidia glandulosa v1.0 | Exigl | Nagy LG et al., 2016 |
| Fibroporia radiculosa TFFH 294 | Fibra | Tang JD et al., 2012 |
| Fibulorhizoctonia sp. CBS 109695 v1.0 | Fibsp | Nagy LG et al., 2016 |
| Fistulina hepatica v1.0 | Fishe | Floudas D et al 2015 |
| Fomitiporia mediterranea v1.0 | Fomme | Floudas D et al., 2012 |
| Fomitopsis pinicola FP-58527 SS1 v3.0 | Fompi | Floudas D et al., 2012 |
| Galerina marginata v1.0 | Galma | Riley R et al., 2014 |
| Gloeophyllum trabeum v1.0 | Glotr | Floudas D et al., 2012 |
| Gonapodya prolifera v1.0 | Ganpr | Chang Y et al., 2015 |
| Heterobasidion annosum v2.0 | Hetan | Olson A et al., 2012 |
| Hypholoma sublateritium v1.0 | Hypsu | Kohler A et al., 2015 |
| Jaapia argillacea v1.0 | Jaaar | Riley R et al., 2014 |
| Laccaria amethystina LaAM-08-1 v1.0 | Lacam | Kohler A et al., 2015 |
| Laccaria bicolor v2.0 | Lacbi | Martin F et al., 2008 |
| Laetiporus sulphureus var. sulphureus v1.0 | Laesu | Nagy LG et al., 2016 |
| Malassezia sympodialis ATCC 42132 | Malsy | Gioti A et al., 2013 |
| Melampsora laricis-populina v1.0 | Mellp | Duplessis S et al., 2011 |
| Mixia osmundae IAM 14324 v1.0 | Mixos | Toome M et al., 2014 |
| Moniliophthora perniciosa FA553 | Monpe | Mondego JM et al., 2008 |
| Neolentinus lepideus v1.0 | Neole | Nagy LG et al., 2016 |
| Omphalotus olearius | Ompol | Wawrzyn GT et al., 2012 |
| Paxillus involutus ATCC 200175 v1.0 | Paxin | Kohler A et al., 2015 |
| Paxillus rubicundulus Ve08.2h10 v1.0 | Paxru | Kohler A et al., 2015 |
| Phanerochaete carnosa HHB-10118-Sp v1.0 | Phaca | Suzuki H et al., 2012 |
| Phanerochaete chrysosporium RP-78 v2.2 | Phchr | Ohm RA et al., 2014 |

| | | |
|---|---|---|
| Phlebia brevispora HHB-7030 SS6 v1.0 | Phlbr | Binder M et al., 2013 |
| Phlebiopsis gigantea v1.0 | Phlgi | Hori et al., 2014 |
| Piloderma croceum F 1598 v1.0 | Pilcr | Kohler A et al., 2015 |
| Piriformospora indica DSM 11827 from MPI | Pirin | Zuccaro A et al., 2011 |
| Pisolithus microcarpus 441 v1.0 | Pismi | Kohler A et al., 2015 |
| Pisolithus tinctorius Marx 270 v1.0 | Pisti | Kohler A et al., 2015 |
| Pleurotus ostreatus PC15 v2.0 | Pleos | Riley R et al., 2014 |
| Plicaturopsis crispa v1.0 | Plicr | Kohler A et al., 2015 |
| Postia placenta MAD 698-R v1.0 | Pospl | Martinez D et al., 2009 |
| Pseudozyma antarctica T-34 | Psean | Morita T et al., 2013 |
| Pseudozyma hubeiensis SY62 | Psehu | Konishi M et al., 2013 |
| Puccinia graminis | puccgr | Duplessis S et al., 2011 |
| Puccinia striiformis f. sp. tritici PST-130 | Pucst | Cantu D et al., 2011 |
| Punctularia strigosozonata v1.0 | Punst | Floudas D et al., 2012 |
| Pycnoporus cinnabarinus BRFM 137 | Pycci | Levasseur A et al., 2014 |
| Rhizoctonia solani AG-1 IB | Rhiso | Wibberg D et al., 2013 |
| Rickenella mellea v1.0 | Ricme | Nagy LG et al., 2016 |
| Schizopora paradoxa KUC8140 v1.0 | Schpa | Min et al., 2015 |
| Scleroderma citrinum Foug A v1.0 | Sclci | Kohler A et al., 2015 |
| Sebacina vermifera MAFF 305830 v1.0 | Sebve | Kohler A et al., 2015 |
| Serpula lacrymans S7.9 v2.0 | Serla | Eastwood DC et al., 2011 |
| Sistotremastrum niveocremeum HHB9708 ss-1 1.0 | Sisni | Nagy LG et al., 2016 |
| Sistotremastrum suecicum v1.0 | Sissu | Nagy LG et al., 2016 |
| Sphaerobolus stellatus v1.0 | Sphst | Kohler A et al., 2015 |
| Sporisorium reilianum SRZ2 | Spore | Schirawski J et al., 2010 |
| Stereum hirsutum FP-91666 SS1 v1.0 | Stehi | Floudas D et al., 2012 |
| Suillus brevipes v1.0 | Suibr | Branco S et al., 2015 |
| Suillus luteus UH-Slu-Lm8-n1 v1.0 | Suilu | Kohler A et al., 2015 |
| Tilletiaria anomala UBC 951 v1.0 | Tilan | Toome M et al., 2014 |
| Trametes versicolor v1.0 | Trave | Floudas D et al., 2012 |
| Tremella mesenterica Fries v1.0 | Treme | Floudas D et al., 2012 |
| Trichosporon oleaginosus IBC0246 v1.0 | Triol | Kourist et al., 2015 |
| Tulasnella calospora AL13/4D v1.0 | Tulca | Kohler A et al., 2015 |
| Ustilago maydis | Ustma | Kamper J et al., 2006 |
| Volvariella volvacea V23 | Volvo | Bao D et al., 2013 |
| Wolfiporia cocos MD-104 SS10 v1.0 | Wolco | Floudas D et al., 2012 |
| **Zygomycota** | | |
| Coemansia reversa NRRL 1564 v1.0 | Coere | Chang Y et al., 2015 |
| Conidiobolus coronatus NRRL28638 v1.0 | Conco | Chang Y et al., 2015 |
| Ganoderma sp. 10597 SS1 v1.0 | Gansp | Binder M et al., 2013 |
| Lichtheimia corymbifera | Licht | Schwartze et al., 2014 |
| Mucor circinneloides | Mucor | Corrochano LM et al., 2016 |
| Orpinomyces sp. | Orpsp | Youssef NH et al., 2013 |

| | | |
|---|---|---|
| Phycomyces blakesleeanus NRRL1555 v2.0 | Phyco | Corrochano LM et al., 2016 |
| Rhizophagus irregularis DAOM 181602 v1.0 | Gloin | Tisserant E et al., 2013 |
| Rhizopus microsporus var. chinensis CCTCC M201020 | Rhich | Wang D et al., 2013 |
| Rhizopus oryzae 99-880 from Broad | Rhior | Ma LJ et al., 2009 |
| Syncephalastrum racemosum NRRL 2496 v1.0 | Synrac | http://genome.jgi.doe.gov/Synrac1/Synrac1.home.html |
| **Microsporidia** | | |
| Antonospora locustae HM-2013 | Antlo | Slamovits CH et al., 2004; Corradi et al., 2007 |
| Encephalitozoon cuniculi GB-M1 | Enccu | Peyretaillade E et al., 2009; Katinka et al., 2001 |
| Encephalitozoon hellem ATCC 50504 | Enche | Pombert JF et al., 2012 |
| Encephalitozoon intestinalis ATCC 50506 | Encin | Corradi N et al., 2010 |
| Encephalitozoon romaleae SJ-2008 | Encro | Pombert JF et al., 2012 |
| Enterocytozoon bieneusi H348 | Entbi | Akiyoshi DE et al., 2009 |
| Nematocida parisii ERTm1 | Nempa | Cuomo CA et al., 2012 |
| Nosema ceranae BRL01 | Nosce | Cornman RS et al., 2009, Chen L et al., 2009 |
| Rozella allomyces (Cryptomycota) | Rosal | James TZ et al., 2013 |

## 2 References for fungal genomes

Akiyoshi DE, Morrison HG, Lei S, Feng X, Zhang Q, Corradi N, et al. Genomic survey of the non-cultivatable opportunistic human pathogen, Enterocytozoon bieneusi. PLoS Pathog. 2009 Jan;5(1):e1000261. doi: 10.1371/journal.ppat.1000261.

Amselem J, Cuomo CA, van Kan JA, Viaud M, Benito EP, Couloux A, et al. Genomic analysis of the necrotrophic fungal pathogens Sclerotinia sclerotiorum and Botrytis cinerea. PLoS Genet. 2011 Aug;7(8):e1002230. doi: 10.1371/journal.pgen.1002230.

Arnaud MB, Cerqueira GC, Inglis DO, Skrzypek MS, Binkley J, Chibucos MC, et al. The Aspergillus Genome Database (AspGD): recent developments in comprehensive multispecies curation, comparative genomics and community resources. Nucleic Acids Res. 2012 Jan;40(Database issue):D653-9. doi: 10.1093/nar/gkr875

Bao D, Gong M, Zheng H, Chen M, Zhang L, Wang H, et al. Sequencing and comparative analysis of the straw mushroom (Volvariella volvacea) genome. PLoS One. 2013;8(3):e58294. doi: 10.1371/journal.pone.0058294.

Berka RM, Grigoriev IV, Otillar R, Salamov A, Grimwood J, Reid I, et al. Comparative genomic analysis of the thermophilic biomass-degrading fungi Myceliophthora thermophila and Thielavia terrestris. Nat Biotechnol. 2011 Oct 2;29(10):922-7. doi: 10.1038/nbt.1976.

Binder M, Justo A, Riley R, Salamov A, Lopez-Giraldez F, Sjokvist E, et al. Phylogenetic and phylogenomic overview of the Polyporales. Mycologia. 2013 Nov-Dec;105(6):1350-73. doi: 10.3852/13-003.

Blanco-Ulate B, Rolshausen PE, Cantu D Draft Genome Sequence of the Grapevine Dieback Fungus Eutypa lata UCR-EL1. Genome Announc. 2013 May 30;1(3). pii: e00228-13. doi: 10.1128/genomeA.00228-13

Branco S, Gladieux P, Ellison CE, Kuo A, LaButti K, Lipzen A, et al. Genetic isolation between two recently diverged populations of a symbiotic fungus. Mol Ecol. 2015 Jun;24(11):2747-58. doi: 10.1111/mec.13132.

Burmester A, Shelest E, Glockner G, Heddergott C, Schindler S, Staib P, et al. Comparative and functional genomics provide insights into the pathogenicity of dermatophytic fungi. Genome Biol. 2011;12(1):R7. doi: 10.1186/gb-2011-12-1-r7.

Cantu D, Govindarajulu M, Kozik A, Wang M, Chen X, Kojima KK, et al. Next generation sequencing provides rapid access to the genome of Puccinia striiformis f. sp. tritici, the causal agent of wheat stripe rust. PLoS One. 2011;6(8):e24230. doi: 10.1371/journal.pone.0024230

Chang Y, Wang S, Sekimoto S, Aerts AL, Choi C, Clum A, et al. (2015) Phylogenomic Analyses Indicate that Early Fungi Evolved Digesting Cell Walls of Algal Ancestors of Land Plants Genome Biol Evol 14: 1590-1601 doi: 10.1093/gbe/evv090

Chen L, Yue Q, Zhang X, Xiang M, Wang C, Li S, et al. Genomics-driven discovery of the pneumocandin biosynthetic gene cluster in the fungus Glarea lozoyensis. BMC Genomics. 2013 May 20;14:339. doi: 10.1186/1471-2164-14-339.

Chen YP, Evans JD, Murphy C, Gutell R, Zuker M, Gundensen-Rindal D, et al. Morphological, molecular, and phylogenetic characterization of Nosema ceranae, a microsporidian parasite isolated from the European honey bee, Apis mellifera. J Eukaryot Microbiol. 2009 Mar-Apr;56(2):142-7. doi:10.1111/j.1550-7408.2008.00374.x.

Cisse OH, Pagni M, Hauser PM De novo assembly of the Pneumocystis jirovecii genome from a single bronchoalveolar lavage fluid specimen from a patient. MBio. 2012 Dec 26;4(1):e00428-12. doi: 10.1128/mBio.00428-12.

Collins C, Keane TM, Turner DJ, O'Keeffe G, Fitzpatrick DA, Doyle S Genomic and proteomic dissection of the ubiquitous plant pathogen, Armillaria mellea: toward a new infection model system. J Proteome Res. 2013 Jun 7;12(6):2552-70. doi: 10.1021/pr301131t.

Condon BJ, Leng Y, Wu D, Bushley KE, Ohm RA, Otillar R, et al. Comparative genome structure, secondary metabolite, and effector coding capacity across Cochliobolus pathogens. PLoS Genet. 2013;9(1):e1003233. doi: 10.1371/journal.pgen.1003233.

Cornman RS, Chen YP, Schatz MC, Street C, Zhao Y, Desany B, et al. Genomic analyses of the microsporidian Nosema ceranae, an emergent pathogen of honey bees. PLoS Pathog. 2009 Jun;5(6):e1000466. doi: 10.1371/journal.ppat.1000466.

Corradi N, Akiyoshi DE, Morrison HG, Feng X, Weiss LM, Tzipori S, et al. Patterns of genome evolution among the microsporidian parasites Encephalitozoon cuniculi, Antonospora locustae and Enterocytozoon bieneusi. PLoS One. 2007 Dec 5;2(12):e1277.

Corradi N, Pombert JF, Farinelli L, Didier ES, Keeling PJ The complete sequence of the smallest known nuclear genome from the microsporidian Encephalitozoon intestinalis. Nat Commun. 2010 Sep 21;1:77. doi: 10.1038/ncomms1082.

Corrochano LM, Kuo A, Marcet-Houben M, Polaino S, Salamov A, Villalobos-Escobedo JM, et al. Expansion of Signal Transduction Pathways in Fungi by Extensive Genome Duplication. Curr Biol. 2016 Jun 20;26(12):1577-84. doi: 10.1016/j.cub.2016.04.038.

Cuomo CA, Desjardins CA, Bakowski MA, Goldberg J, Ma AT, Becnel JJ, et al. Microsporidian genome analysis reveals evolutionary strategies for obligate intracellular growth. Genome Res. 2012 Dec;22(12):2478-88. doi: 10.1101/gr.142802.112

Cuomo CA, Guldener U, Xu JR, Trail F, Turgeon BG, Di Pietro A, et al. The Fusarium graminearum genome reveals a link between localized polymorphism and pathogen specialization. Science. 2007 Sep 7;317(5843):1400-2.

de Wit PJ, van der Burgt A, Okmen B, Stergiopoulos I, Abd-Elsalam KA, Aerts AL, et al. The genomes of the fungal plant pathogens Cladosporium fulvum and Dothistroma septosporum reveal adaptation to different hosts and lifestyles but also signatures of common ancestry. PLoS Genet. 2012;8(11):e1003088. doi: 10.1371/journal.pgen.1003088.

Dean RA, Talbot NJ, Ebbole DJ, Farman ML, Mitchell TK, Orbach MJ, Thon M, Kulkarni R, Xu JR, Pan H, Read ND, Lee YH, Carbone I, Brown D, Oh YY, Donofrio N, Jeong JS, Soanes DM, Djonovic S, Kolomiets E, Rehmeyer C, Li W, Harding M, Kim S, Lebrun MH, Bohnert H, Coughlan S, Butler J, Calvo S, Ma LJ, Nicol R, Purcell S, Nusbaum C, Galagan JE, Birren BW The genome sequence of the rice blast fungus Magnaporthe grisea. Nature. 2005 Apr 21;434(7036):980-6.

Desjardins CA, Champion MD, Holder JW, Muszewska A, Goldberg J, Bailao AM, et al. Comparative genomic analysis of human fungal pathogens causing paracoccidioidomycosis. PLoS Genet. 2011 Oct;7(10):e1002345. doi: 10.1371/journal.pgen.1002345.

Dhillon B, Feau N, Aerts AL, Beauseigle S, Bernier L, Copeland A, Foster A, Gill N, Henrissat B, Herath P, LaButti KM, Levasseur A, Lindquist EA, Majoor E, Ohm RA, Pangilinan JL, Pribowo A, Saddler JN, Sakalidis ML, de Vries RP, Grigoriev IV, Goodwin SB, Tanguay P, Hamelin RC Horizontal gene transfer and gene dosage drives adaptation to wood colonization in a tree pathogen. Proc Natl Acad Sci U S A. 2015 Mar 17;112(11):3451-6. doi: 10.1073/pnas.1424293112.

Dietrich FS, Voegeli S, Brachat S, Lerch A, Gates K, Steiner S, et al. The Ashbya gossypii genome as a tool for mapping the ancient Saccharomyces cerevisiae genome. Science. 2004 Apr 9;304(5668):304-7. Epub 2004 Mar 4.

DiGuistini S, Wang Y, Liao NY, Taylor G, Tanguay P, Feau N, Henrissat B, Chan SK, Hesse-Orce U, Alamouti SM, Tsui CK, Docking RT, Levasseur A, Haridas S, Robertson G, Birol I, Holt RA, Marra MA, Hamelin RC, Hirst M, Jones SJ, Bohlmann J, Breuil C Genome and transcriptome analyses of the mountain pine beetle-fungal symbiont Grosmannia clavigera, a lodgepole pine pathogen. Proc Natl Acad Sci U S A. 2011 Feb 8;108(6):2504-9. doi: 10.1073/pnas.1011289108.

Dujon B, Sherman D, Fischer G, Durrens P, Casaregola S, Lafontaine I, et al. Genome evolution in yeasts. Nature. 2004 Jul 1;430(6995):35-44.

Duplessis S, Cuomo CA, Lin YC, Aerts A, Tisserant E, Veneault-Fourrey C, et al. Obligate biotrophy features unraveled by the genomic analysis of rust fungi. Proc Natl Acad Sci U S A. 2011 May 31;108(22):9166-71. doi: 10.1073/pnas.1019315108.

Eastwood DC, Floudas D, Binder M, Majcherczyk A, Schneider P, Aerts A, et al. The plant cell wall-decomposing machinery underlies the functional diversity of forest fungi. Science. 2011 Aug 5;333(6043):762-5. doi: 10.1126/science.1205411.

Ellwood SR, Liu Z, Syme RA, Lai Z, Hane JK, Keiper F, et al. A first genome assembly of the barley fungal pathogen Pyrenophora teres f. teres. Genome Biol. 2010;11(11):R109. doi: 10.1186/gb-2010-11-11-r109.

Espagne E, Lespinet O, Malagnac F, Da Silva C, Jaillon O, Porcel BM, et al. The genome sequence of the model ascomycete fungus Podospora anserina. Genome Biol. 2008;9(5):R77. doi: 10.1186/gb-2008-9-5-r77.

Fedorova ND, Khaldi N, Joardar VS, Maiti R, Amedeo P, Anderson MJ, et al. Genomic islands in the pathogenic filamentous fungus Aspergillus fumigatus. PLoS Genet. 2008 Apr 11;4(4):e1000046. doi: 10.1371/journal.pgen.1000046.

Floudas D, Binder M, Riley R, Barry K, Blanchette RA, Henrissat B, et al. (2012) The Paleozoic origin of enzymatic lignin decomposition reconstructed from 31 fungal genomes. Science. 336(6089):1715-1719.

Floudas D, Held BW, Riley R, Nagy LG, Koehler G, Ransdell AS, et al. Evolution of novel wood decay mechanisms in Agaricales revealed by the genome sequences of Fistulina hepatica and Cylindrobasidium torrendii. Fungal Genet Biol. 2015 Mar;76:78-92. doi: 10.1016/j.fgb.2015.02.002.

Futagami T, Mori K, Yamashita A, Wada S, Kajiwara Y, Takashita H, et al. Genome sequence of the white koji mold Aspergillus kawachii IFO 4308, used for brewing the Japanese distilled spirit shochu. Eukaryot Cell. 2011 Nov;10(11):1586-7. doi: 10.1128/EC.05224-11.

Galagan JE, Calvo SE, Borkovich KA, Selker EU, Read ND, Jaffe D, et al. The genome sequence of the filamentous fungus Neurospora crassa. Nature. 2003 Apr 24;422(6934):859-68

Gao Q, Jin K, Ying SH, Zhang Y, Xiao G, Shang Y, et al. Genome sequencing and comparative transcriptomics of the model entomopathogenic fungi Metarhizium anisopliae and M. acridum. PLoS Genet. 2011 Jan 6;7(1):e1001264. doi: 10.1371/journal.pgen.1001264.

Gattiker A, Rischatsch R, Demougin P, Voegeli S, Dietrich FS, Philippsen P, et al. Ashbya Genome Database 3.0: a cross-species genome and transcriptome browser for yeast biologists. BMC Genomics. 2007 Jan 9;8:9.

Gianoulis TA, Griffin MA, Spakowicz DJ, Dunican BF, Alpha CJ, Sboner A, et al. Genomic analysis of the hydrocarbon-producing, cellulolytic, endophytic fungus Ascocoryne sarcoides. PLoS Genet. 2012;8(3):e1002558. doi: 10.1371/journal.pgen.1002558.

Gioti A, Nystedt B, Li W, Xu J, Andersson A, Averette AF, et al. Genomic insights into the atopic eczema-associated skin commensal yeast Malassezia sympodialis. MBio. 2013 Jan 22;4(1):e00572-12. doi: 10.1128/mBio.00572-12

Goffeau A, Barrell BG, Bussey H, Davis RW, Dujon B, Feldmann H, et al. Life with 6000 genes. Science. 1996 Oct 25;274(5287):546, 563-7.

Gostincar C, Ohm RA, Kogej T, Sonjak S, Turk M, Zajc J, Zalar P, Grube M, Sun H, Han J, Sharma A, Chiniquy J, Ngan CY, Lipzen A, Barry K, Grigoriev IV, Gunde-Cimerman N Genome sequencing of four Aureobasidium pullulans varieties: biotechnological potential, stress tolerance, and description of new species. BMC Genomics. 2014 Jul 1;15:549. doi: 10.1186/1471-2164-15-549.

Hane JK, Lowe RG, Solomon PS, Tan KC, Schoch CL, Spatafora JW, et al. Dothideomycete plant interactions illuminated by genome sequencing and EST analysis of the wheat pathogen Stagonospora nodorum. Plant Cell. 2007 Nov;19(11):3347-68.

Haridas S, Wang Y, Lim L, Massoumi Alamouti S, Jackman S, Docking R, et al. The genome and transcriptome of the pine saprophyte Ophiostoma piceae, and a comparison with the bark beetle-associated pine pathogen Grosmannia clavigera. BMC Genomics. 2013 Jun 2;14:373. doi: 10.1186/1471-2164-14-373.

Hori C, Ishida T, Igarashi K, Samejima M, Suzuki H, Master E, et al. Analysis of the Phlebiopsis gigantea genome, transcriptome and secretome provides insight into its pioneer colonization strategies of wood. PLoS Genet. 2014 Dec 4;10(12):e1004759. doi: 10.1371/journal.pgen.1004759.

Islam MS, Haque MS, Islam MM, Emdad EM, Halim A, Hossen QM, et al. Tools to kill: genome of one of the most destructive plant pathogenic fungi Macrophomina phaseolina. BMC Genomics. 2012 Sep 19;13:493. doi: 10.1186/1471-2164-13-493.

James TY, Pelin A, Bonen L, Ahrendt S, Sain D, Corradi N, Stajich JE Shared signatures of parasitism and phylogenomics unite Cryptomycota and microsporidia. Curr Biol. 2013 Aug 19;23(16):1548-53. doi: 10.1016/j.cub.2013.06.057.

Kamper J, Kahmann R, Bolker M, Ma LJ, Brefort T, Saville BJ, et al. Insights from the genome of the biotrophic fungal plant pathogen Ustilago maydis. Nature. 2006 Nov 2;444(7115):97-101.

Katinka MD, Duprat S, Cornillot E, Metenier G, Thomarat F, Prensier G, et al. Genome sequence and gene compaction of the eukaryote parasite Encephalitozoon cuniculi. Nature. 2001 Nov 22;414(6862):450-3.

Kis-Papo T, Weig AR, Riley R, Persoh D, Salamov A, Sun H, et al. Genomic adaptations of the halophilic Dead Sea filamentous fungus Eurotium rubrum. Nat Commun. 2014 May 9;5:3745. doi: 10.1038/ncomms4745.

Klosterman SJ, Subbarao KV, Kang S, Veronese P, Gold SE, Thomma BP, et al. Comparative genomics yields insights into niche adaptation of plant vascular wilt pathogens. PLoS Pathog. 2011 Jul;7(7):e1002137. doi: 10.1371/journal.ppat.1002137. Epub 2011 Jul 28.

Kohler A, Kuo A, Nagy LG, Morin E, Barry KW, Buscot F, et al. Convergent losses of decay mechanisms and rapid turnover of symbiosis genes in mycorrhizal mutualists. Nat Genet. 2015 Apr;47(4):410-5. doi: 10.1038/ng.3223.

Konishi M, Hatada Y, Horiuchi J Draft Genome Sequence of the Basidiomycetous Yeast-Like Fungus Pseudozyma hubeiensis SY62, Which Produces an Abundant Amount of the Biosurfactant Mannosylerythritol Lipids. Genome Announc. 2013 Jun 27;1(4). pii: e00409-13. doi: 10.1128/genomeA.00409-13.

Kourist R, Bracharz F, Lorenzen J, Kracht ON, Chovatia M, Daum C, et al. Genomics and Transcriptomics Analyses of the Oil-Accumulating Basidiomycete Yeast Trichosporon oleaginosus: Insights into Substrate Utilization and Alternative Evolutionary Trajectories of Fungal Mating Systems. MBio. 2015 Jul 21;6(4):e00918. doi: 10.1128/mBio.00918-15.

Kubicek CP, Herrera-Estrella A, Seidl-Seiboth V, Martinez DA, Druzhinina IS, Thon M, et al. CM, Monte E, Baker SE, Grigoriev IV Comparative genome sequence analysis underscores mycoparasitism as the ancestral life style of Trichoderma. Genome Biol. 2011;12(4):R40. doi: 10.1186/gb-2011-12-4-r40.

Levasseur A, Lomascolo A, Chabrol O, Ruiz-Duenas FJ, Boukhris-Uzan E, Piumi F, et al. 2014. The genome of the white-rot fungus Pycnoporus cinnabarinus: a basidiomycete model with a versatile arsenal for lignocellulosic biomass breakdown. BMC Genomics 15:486. doi:.10.1186/1471-2164-15-486

Liu G, Zhang L, Wei X, Zou G, Qin Y, Ma L, et al. Genomic and secretomic analyses reveal unique features of the lignocellulolytic enzyme system of Penicillium decumbens. PLoS One. 2013;8(2):e55185. doi: 10.1371/journal.pone.0055185.

Loftus BJ, Fung E, Roncaglia P, Rowley D, Amedeo P, Bruno D, et al. The genome of the basidiomycetous yeast and human pathogen Cryptococcus neoformans. Science. 2005 Feb 25;307(5713):1321-4.

Ma LJ, Ibrahim AS, Skory C, Grabherr MG, Burger G, Butler M, et al. Genomic analysis of the basal lineage fungus Rhizopus oryzae reveals a whole-genome duplication. PLoS Genet. 2009 Jul;5(7):e1000549. doi: 10.1371/journal.pgen.1000549.

Ma LJ, van der Does HC, Borkovich KA, Coleman JJ, Daboussi MJ, Di Pietro A, et al. Comparative genomics reveals mobile pathogenicity chromosomes in Fusarium. Nature. 2010 Mar 18;464(7287):367-73. doi: 10.1038/nature08850.

Marcet-Houben M, Ballester AR, de la Fuente B, Harries E, Marcos JF, Gonzalez-Candelas L, et al. Genome sequence of the necrotrophic fungus Penicillium digitatum, the main postharvest pathogen of citrus. BMC Genomics. 2012 Nov 21;13:646. doi: 10.1186/1471-2164-13-646.

Marsberg A1, Kemler M1, Jami F2, Nagel JH1, Postma-Smidt A3, Naidoo S1, et al. Botryosphaeria dothidea: A latent pathogen of global importance to woody plant health. Mol Plant Pathol. 2016 Sep 28. doi: 10.1111/mpp.12495.

Martin F, Aerts A, Ahren D, Brun A, Danchin EG, Duchaussoy F, et al. The genome of Laccaria bicolor provides insights into mycorrhizal symbiosis. Nature. 2008 Mar 6;452(7183):88-92. doi: 10.1038/nature06556.

Martin F, Kohler A, Murat C, Balestrini R, Coutinho PM, Jaillon O, et al. Perigord black truffle genome uncovers evolutionary origins and mechanisms of symbiosis. Nature. 2010 Apr 15;464(7291):1033-8. doi: 10.1038/nature08867.

Martinez D, Berka RM, Henrissat B, Saloheimo M, Arvas M, Baker SE, et al. Genome sequencing and analysis of the biomass-degrading fungus Trichoderma reesei (syn. Hypocrea jecorina). Nat Biotechnol. 2008 May;26(5):553-60. doi: 10.1038/nbt1403.

Martinez D, Challacombe J, Morgenstern I, Hibbett D, Schmoll M, Kubicek CP, et al. Genome, transcriptome, and secretome analysis of wood decay fungus Postia placenta supports unique mechanisms of lignocellulose conversion. Proc Natl Acad Sci U S A. 2009 Feb 10;106(6):1954-9. doi: 10.1073/pnas.0809575106.

Meerupati T, Andersson KM, Friman E, Kumar D, Tunlid A, Ahren D Genomic mechanisms accounting for the adaptation to parasitism in nematode-trapping fungi. PLoS Genet. 2013 Nov;9(11):e1003909. doi: 10.1371/journal.pgen.1003909

Min B, Park H, Jang Y, Kim JJ, Kim KH, Pangilinan J, et al. Genome sequence of a white rot fungus Schizopora paradoxa KUC8140 for wood decay and mycoremediation. J Biotechnol. 2015 Oct 10;211:42-3. doi: 10.1016/j.jbiotec.2015.06.426.

Mondego JM, Carazzolle MF, Costa GG, Formighieri EF, Parizzi LP, Rincones J, et al. A genome survey of Moniliophthora perniciosa gives new insights into Witches' Broom Disease of cacao. BMC Genomics. 2008 Nov 18;9:548. doi: 10.1186/1471-2164-9-548

Morita T, Koike H, Koyama Y, Hagiwara H, Ito E, Fukuoka T, et al. Genome Sequence of the Basidiomycetous Yeast Pseudozyma antarctica T-34, a Producer of the Glycolipid Biosurfactants Mannosylerythritol Lipids. Genome Announc. 2013 Apr 4;1(2):e0006413. doi: 10.1128/genomeA.00064-13.

Nagy LG, Riley R, Tritt A, Adam C, Daum C, Floudas D, et al. Comparative Genomics of Early-Diverging Mushroom-Forming Fungi Provides Insights into the Origins of Lignocellulose Decay Capabilities. Mol Biol Evol. 2016 Apr;33(4):959-70. doi: 10.1093/molbev/msv337.

Nierman W.C., Fedorova-Abrams N.D., Andrianopoulos A. Genome sequence of the AIDS-associated pathogen Penicillium marneffei (ATCC18224) and its near taxonomic relative Talaromyces stipitatus (ATCC10500). Genome Announc. 2015:E0155914-E0155914(2015)

Nierman WC, Pain A, Anderson MJ, Wortman JR, Kim HS, Arroyo J, et al. Genomic sequence of the pathogenic and allergenic filamentous fungus Aspergillus fumigatus. Nature. 2005 Dec 22;438(7071):1151-6.

O'Connell RJ, Thon MR, Hacquard S, Amyotte SG, Kleemann J, Torres MF, et al. Lifestyle transitions in plant pathogenic Colletotrichum fungi deciphered by genome and transcriptome analyses. Nat Genet. 2012 Sep;44(9):1060-5. doi: 10.1038/ng.2372.

Ohm RA, Feau N, Henrissat B, Schoch CL, Horwitz BA, Barry KW, et al. Diverse lifestyles and strategies of plant pathogenesis encoded in the genomes of eighteen Dothideomycetes fungi. PLoS Pathog. 2012;8(12):e1003037. doi: 10.1371/journal.ppat.1003037.

Ohm RA, Riley R, Salamov A, Min B, Choi IG, Grigoriev IV Genomics of wood-degrading fungi. Fungal Genet Biol. 2014 Nov;72:82-90. doi: 10.1016/j.fgb.2014.05.001.

Olson A, Aerts A, Asiegbu F, Belbahri L, Bouzid O, Broberg A, et al. Insight into trade-off between wood decay and parasitism from the genome of a fungal forest pathogen. New Phytol. 2012 Jun;194(4):1001-13. doi: 10.1111/j.1469-8137.2012.04128.x.

Pel HJ1, de Winde JH, Archer DB, Dyer PS, Hofmann G, Schaap PJ, et al. Genome sequencing and analysis of the versatile cell factory Aspergillus niger CBS 513.88 Nat Biotechnol. 2007 Feb;25(2):221-31.

Peter M, Kohler A, Ohm RA, Kuo A, Krutzmann J, Morin E, et al. Ectomycorrhizal ecology is imprinted in the genome of the dominant symbiotic fungus Cenococcum geophilum. Nat Commun. 2016 Sep 7;7:12662. doi: 10.1038/ncomms12662

Peyretaillade E, Goncalves O, Terrat S, Dugat-Bony E, Wincker P, Cornman RS, et al. Identification of transcriptional signals in Encephalitozoon cuniculi widespread among Microsporidia phylum: support for accurate structural genome annotation. BMC Genomics. 2009 Dec 15;10:607. doi: 10.1186/1471-2164-10-607

Piskur J, Ling Z, Marcet-Houben M, Ishchuk OP, Aerts A, LaButti K, et al. The genome of wine yeast Dekkera bruxellensis provides a tool to explore its food-related properties. Int J Food Microbiol. 2012 Jul 2;157(2):202-9. doi: 10.1016/j.ijfoodmicro.2012.05.008.

Pombert JF, Selman M, Burki F, Bardell FT, Farinelli L, Solter LF, et al. Gain and loss of multiple functionally related, horizontally transferred genes in the reduced genomes of two microsporidian parasites. Proc Natl Acad Sci U S A. 2012 Jul 31;109(31):12638-43. doi: 10.1073/pnas.1205020109.

Rhind N, Chen Z, Yassour M, Thompson DA, Haas BJ, Habib N, et al. Comparative functional genomics of the fission yeasts. Science. 2011 May 20;332(6032):930-6. doi: 10.1126/science.1203357.

Riley R, Haridas S, Wolfe KH, Lopes MR, Hittinger CT, Goker M, et al. Comparative genomics of biotechnologically important yeasts. Proc Natl Acad Sci U S A. 2016 Aug 30;113(35):9882-7. doi: 10.1073/pnas.1603941113.

Riley R, Salamov AA, Brown DW, Nagy LG, Floudas D, Held BW, et al. Extensive sampling of basidiomycete genomes demonstrates inadequacy of the white-rot/brown-rot paradigm for wood decay fungi. Proc Natl Acad Sci U S A. 2014 Jul 8;111(27):9923-8. doi: 10.1073/pnas.1400592111.

Rouxel T, Grandaubert J, Hane JK, Hoede C, van de Wouw AP, Couloux A, et al. Effector diversification within compartments of the Leptosphaeria maculans genome affected by Repeat-Induced Point mutations. Nat Commun. 2011 Feb 15;2:202. doi: 10.1038/ncomms1189.

Sacerdot C, Casaregola S, Lafontaine I, Tekaia F, Dujon B, Ozier-Kalogeropoulos O Promiscuous DNA in the nuclear genomes of hemiascomycetous yeasts. FEMS Yeast Res. 2008 Sep;8(6):846-57. doi: 10.1111/j.1567-1364.2008.00409.x.

Schirawski J, Mannhaupt G, Munch K, Brefort T, Schipper K, Doehlemann G, et al. Pathogenicity determinants in smut fungi revealed by genome comparison. Science. 2010 Dec 10;330(6010):1546-8. doi: 10.1126/science.1195330.

Schwartze VU, Winter S, Shelest E, Marcet-Houben M, Horn F, Wehner S, et al. Gene expansion shapes genome architecture in the human pathogen Lichtheimia corymbifera: an evolutionary genomics analysis in the ancient terrestrial mucorales (Mucoromycotina). PLoS Genet. 2014 Aug 14;10(8):e1004496. doi: 10.1371/journal.pgen.1004496.

Sharpton TJ, Stajich JE, Rounsley SD, Gardner MJ, Wortman JR, Jordar VS, Maiti R, Kodira CD, Neafsey DE, Zeng Q, Hung CY, McMahan C, Muszewska A, Grynberg M, Mandel MA, Kellner EM, Barker BM, Galgiani JN, Orbach MJ, Kirkland TN, Cole GT, Henn MR, Birren BW, Taylor JW Comparative genomic analyses of the human fungal pathogens Coccidioides and their relatives. Genome Res. 2009 Oct;19(10):1722-31. doi: 10.1101/gr.087551.108.

Slamovits CH, Fast NM, Law JS, Keeling PJ Genome compaction and stability in microsporidian intracellular parasites. Curr Biol. 2004 May 25;14(10):891-6.

Spanu PD, Abbott JC, Amselem J, Burgis TA, Soanes DM, Stuber K, et al. Genome expansion and gene loss in powdery mildew fungi reveal tradeoffs in extreme parasitism. Science. 2010 Dec 10;330(6010):1543-6. doi: 10.1126/science.1194573.

Staats M, van Kan JA Genome update of Botrytis cinerea strains B05.10 and T4. Eukaryot Cell. 2012 Nov;11(11):1413-4. doi: 10.1128/EC.00164-12.

Stajich JE, Wilke SK, Ahren D, Au CH, Birren BW, Borodovsky M, et al. Insights into evolution of multicellular fungi from the assembled chromosomes of the mushroom Coprinopsis cinerea (Coprinus cinereus). Proc Natl Acad Sci U S A. 2010 Jun 29;107(26):11889-94. doi: 10.1073/pnas.1003391107.

Suzuki H, MacDonald J, Syed K, Salamov A, Hori C, Aerts A, et al. Comparative genomics of the white-rot fungi, Phanerochaete carnosa and P. chrysosporium, to elucidate the genetic basis of the distinct wood types they colonize. BMC Genomics. 2012 Sep 2;13:444. doi: 10.1186/1471-2164-13-444.

Tang JD, Perkins AD, Sonstegard TS, Schroeder SG, Burgess SC, Diehl SV Short-read sequencing for genomic analysis of the brown rot fungus Fibroporia radiculosa. Appl Environ Microbiol. 2012 Apr;78(7):2272-81. doi: 10.1128/AEM.06745-11.

Tisserant E, Malbreil M, Kuo A, Köhler A, Symeonidi A, Balestrini R, et al. (2013) Genome of an arbuscular mycorrhizal fungus provides insight into the oldest plant symbiosis. Proc Natl Acad Sci U S A 110: 20117-20122.

Toome M, Kuo A, Henrissat B, Lipzen A, Tritt A, Yoshinaga Y, et al.  Draft Genome Sequence of a Rare Smut Relative, Tilletiaria anomala UBC 951. Genome Announc. 2014 Jun 12;2(3). pii: e00539-14. doi: 10.1128/genomeA.00539-14.

Traeger S, Altegoer F, Freitag M, Gabaldon T, Kempken F, Kumar A, et al. The genome and development-dependent transcriptomes of Pyronema confluens: a window into fungal evolution. PLoS Genet. 2013;9(9):e1003820. doi: 10.1371/journal.pgen.1003820.

van den Berg MA, Albang R, Albermann K, Badger JH, Daran JM, Driessen AJ, et al. Genome sequencing and analysis of the filamentous fungus Penicillium chrysogenum. Nat Biotechnol. 2008 Oct;26(10):1161-8. doi: 10.1038/nbt.1498.

Wang D, Wu R, Xu Y, Li M Draft Genome Sequence of Rhizopus chinensis CCTCCM201021, Used for Brewing Traditional Chinese Alcoholic Beverages. Genome Announc. 2013 Feb 28;1(2):e0019512. doi: 10.1128/genomeA.00195-12.

Wawrzyn GT, Quin MB, Choudhary S, Lopez-Gallego F, Schmidt-Dannert C Draft genome of Omphalotus olearius provides a predictive framework for sesquiterpenoid natural product biosynthesis in Basidiomycota. Chem Biol. 2012 Jun 22;19(6):772-83. doi: 10.1016/j.chembiol.2012.05.012.

Wibberg D, Jelonek L, Rupp O, Hennig M, Eikmeyer F, Goesmann A, et al.  Establishment and interpretation of the genome sequence of the phytopathogenic fungus Rhizoctonia solani AG1-IB isolate 7/3/14. J Biotechnol. 2013 Aug 20;167(2):142-55. doi: 10.1016/j.jbiotec.2012.12.010.

Wiemann P, Sieber CM, von Bargen KW, Studt L, Niehaus EM, Espino JJ, et al. Deciphering the cryptic genome: genome-wide analyses of the rice pathogen Fusarium fujikuroi reveal complex regulation of secondary metabolism and novel metabolites. PLoS Pathog. 2013;9(6):e1003475. doi: 10.1371/journal.ppat.1003475

Wohlbach DJ, Kuo A, Sato TK, Potts KM, Salamov AA, Labutti KM, et al.  Comparative genomics of xylose-fermenting fungi for enhanced biofuel production. Proc Natl Acad Sci U S A. 2011 Aug 9;108(32):13212-7. doi: 10.1073/pnas.1103039108.

Wood V, Gwilliam R, Rajandream MA, Lyne M, Lyne R, Stewart A, et al. The genome sequence of Schizosaccharomyces pombe. Nature. 2002 Feb 21;415(6874):871-80.

Xiao G, Ying SH, Zheng P, Wang ZL, Zhang S, Xie XQ, et al.  Genomic perspectives on the evolution of fungal entomopathogenicity in Beauveria bassiana. Sci Rep. 2012;2:483. doi: 10.1038/srep00483.

Yang J, Wang L, Ji X, Feng Y, Li X, Zou C, Genomic and proteomic analyses of the fungus Arthrobotrys oligospora provide insights into nematode-trap formation. PLoS Pathog. 2011 Sep;7(9):e1002179. doi: 10.1371/journal.ppat.1002179.

Youssef NH, Couger MB, Struchtemeyer CG, Liggenstoffer AS, Prade RA, Najar FZ, et al. The genome of the anaerobic fungus Orpinomyces sp. strain C1A reveals the unique evolutionary history of a remarkable plant biomass degrader. Appl Environ Microbiol. 2013 Aug;79(15):4620-34. doi: 10.1128/AEM.00821-13.

Zhaotao Liu, Sen Lian, Baohua Li, Hongyun Lu, Xiangli Dong, and Caixia Wanga Draft Genome Sequence of Botryosphaeria dothidea, the Pathogen of Apple Ring Rot Genome Announc. 2016 Sep-Oct; 4(5): e01142-16. Published online 2016 Oct 27. doi: 10.1128/genomeA.01142-16

Zheng P, Xia Y, Xiao G, Xiong C, Hu X, Zhang S, et al. Genome sequence of the insect pathogenic fungus Cordyceps militaris, a valued traditional Chinese medicine. Genome Biol. 2011 Nov 23;12(11):R116. doi: 10.1186/gb-2011-12-11-r116.

Zuccaro A, Lahrmann U, Guldener U, Langen G, Pfiffi S, Biedenkopf D, et al. Endophytic life strategies decoded by genome and transcriptome analyses of the mutualistic root symbiont Piriformospora indica. PLoS Pathog. 2011 Oct;7(10):e1002290. doi: 10.1371/journal.ppat.1002290.
