# Supplementary material for: Transcription Factors in Fungi: TFome Dynamics, Three Major Families, and Dual-Specificity TFs
Source: Front Genet. 2017 May 4;8:53. doi: 10.3389/fgene.2017.00053 (PMC5415576; doi:10.3389/fgene.2017.00053)
Supplement: Table S2 — Major taxonomic assignments of the TFDFs found in fungi and Microsporidia. (A) Rare TFFs (found in 1-5 species); (B) Typically found in fungi. [file Table2.PDF]

Supplementary Material

Article Title Transcription factors in fungi: TFome dynamics, three major families, and dual-specificity TFs

Ekaterina Shelest*

*** Correspondence:** ekaterina.shelest@leibniz-hki.de

Table S2. Major taxonomic assignments of the TF-like DBD families found in fungi and Microsporidia. **A.** TFDFs found in fungi and microsporidia in trace amounts (1-5 species). The second column of the section A shows the taxonomic assignment and number of species (in the parentheses) possessing the found TFDFs. Abbreviations: A - ascomycetes, B - Basidiomycetes, Z - Zygomycetes, C - Chitrids, M - Microsporidia. For details, consult Suppl. Table S3. **B.** TFDFs reliably detected in fungal and microsporidial genomes (>5 TFs per species).

IPR ID	Domain name		Found in*
**A. Rare DBDFs (found in 1-5 species)**			
IPR004210	BESS motif	A(4),B(1)	Metazoa
IPR011539	Rel homology	A(3),B(2)	Metazoa
IPR013019	MAD homology, MH1	A(2)	Metazoa
IPR013854	Transcription factor AP-2, C-terminal	B(1)	Metazoa
IPR001628	Zinc finger, nuclear hormone receptor-type	Z(2)	Metazoa
IPR013524	Acute myeloid leukemia 1 (AML 1)/Runt	B(1)	Metazoa
IPR009508	Churchill	C(1)	Metazoa
IPR009638	Fez1	A(1)	Metazoa
IPR003350	Homeodomain protein CUT	B(1)	Metazoa
IPR002546	Myogenic basic muscle-specific protein	B(1)	Metazoa
IPR000770	SAND	B(1)	Metazoa+Viridiplantae
IPR005735	Zinc finger, LSD1-type	A(4)	Viridiplantae, plant-spec
IPR003441	No apical meristem (NAM) protein	A(2),B(3)	Viridiplantae
IPR003340	Transcriptional factor B3	A(1)	Viridiplantae, plant-spec
IPR007050	HTH_10, Bacterio-opsin activator	A(1)	Archea
IPR002831	Transcriptional regulator TrmB	A(1)	Bacteria/ Archea
IPR006199	LexA DNA-binding region	A(3),B(2)	Bacteria
IPR000524	Bacterial regulatory protein GntR, HTH	A(1),Z(1),M(2)	Bacteria
IPR000847	Bacterial regulatory protein, LysR	A(2),Z(1),M(1)	Bacteria
IPR002481	Ferric-uptake regulator	A(3),Z(1)	Bacteria
IPR007985	Haemolysin expression modulating, HHA	B(2),Z(2)	Bacteria
IPR013199	HTH Mga	B(2),Z(1)	Bacteria
IPR013196	Helix-turn-helix, type 11	A(1),Z(2)	Bacteria
IPR007911	Flagellar transcriptional activator	A(1),Z(1)	Bacteria
IPR007737	M trans-acting positive regulator	A(1),Z(1)	Bacteria
IPR000831	Trp repressor	A(1),B(1)	Bacteria
IPR002577	Helix-turn-helix, HxlR type	A(1),M(1)	Bacteria
IPR005569	Arc-like DNA binding	Z(1)	Bacteria
IPR010330	Competence-induced protein CoiA-like	A(1)	Bacteria
IPR013198	GTP-sensing helix-turn-helix, CodY, C-terminal	A(1)	Bacteria

IPR010312	GTP-sensing transcriptional pleiotropic repressor CodY, N-terminal	A(1)	Bacteria
IPR000281	HTH_6, Helix-turn-helix protein RpiR	A(1)	Bacteria
IPR007492	LytTr DNA-binding region	A(1)	Bacteria
IPR004356	P pili regulatory PapB protein	Z(1)	Bacteria
IPR005650	Penicillinase repressor	A(1)	Bacteria
IPR001867	Signal transduction response regulator, C-terminal	A(2),Z(1),M(1)	Bacteria
IPR009300	Transcriptional activator RinB	A(1)	Bacteria / Viruses
IPR003176	Viral DNA-binding protein	A(3),B(1),Z(1)	Viruses
IPR003174	Alpha trans-inducing protein (Alpha-TIF)	B(2),Z(2)	Viruses
IPR015241	TF MotA, C-terminal, bacteriophage	B(2),C(1)	Viruses
IPR007031	Poxvirus late transcription factor VLTF3 like	C(2)	Viruses
IPR006834	VITF-3 subunit protein	A(1)	Viruses
**B. DBDFs typically found in fungi (in >5 species)**			
IPR001138	Zn_cluster		FUNGAL+Various eukar groups, 1-5 species in each
IPR003163	APSES		FUNGAL
IPR001083	Copper fist DNA-binding		FUNGAL
IPR006856	Mating-type protein MAT alpha 1		FUNGAL
IPR012340	Nucleic acid-binding, OB-fold		ALL but not true fungi so far: Found in Opisthokonta incertae sedis
IPR001471	Pathogenesis-related transcriptional factor and ERF, DNA-binding		ALL but not metazoans
IPR001387	Helix-turn-helix type 3		ALL but mainly bacteria
IPR007889	Helix-turn-helix, Psq		ALL (eukar+bact)
IPR009057	Homeodomain-like		ALL
IPR009061	Putative DNA binding		ALL
IPR002059	Cold-shock protein, DNA-binding		ALL
IPR018004	KilA, N-terminal/APSES-type HTH, DNA-binding		ALL
IPR009044	ssDNA-binding transcriptional regulator		All eukar (plants)
IPR008895	YL1 nuclear		All eukar (Metazoa)
IPR007604	CP2 transcription factor		Ichthyosporea, Nucleariidae and Fonticulagroup, Choanoflagellida
IPR012294	Transcription factor TFIID, C-terminal/DNA glycosylase, N-terminal		?
IPR005011	SART-1 protein		Eukar-ubiquitous
IPR010770	SGT1		Eukar-ubiquitous
IPR003958	Transcription factor CBF/NF-Y/archaeal histone		Eukar+Archea
IPR000814	TATA-box binding		Eukar+Archea+(Viruses only 3)
IPR004827	Basic-leucine zipper (bZIP) transcription factor		Eukar - various
IPR001289	CCAAT-binding transcription factor, subunit B		Eukar - various
IPR007196	CCR4-Not complex component, Not1		Eukar - various
IPR003150	DNA-binding RFX		Eukar - various
IPR016177	DNA-binding, integrase-type		Eukar - various
IPR001878	Zinc finger, CCHC-type		All eukar+viruses (only 13)
IPR025659	Tubby C-terminal-like domain		All eukar (mostly plants)
IPR007087	Zinc finger, C2H2-type		All eukar
IPR000571	Zinc finger, CCCH-type		All eukar
IPR000679	Zinc finger, GATA-type		All eukar
IPR004823	TATA box binding protein associated factor (TAF)		All eukar
IPR004181	Zinc finger, MIZ-type		All eukar
IPR013932	TATA-binding protein interacting (TIP20)		All eukar
IPR000967	Zinc finger, NF-X1-type		All eukar
IPR010666	Zinc finger, GRF-type		All eukar
IPR003656	Zinc finger, BED-type predicted		All eukar
IPR004198	Zinc finger, C5HC2-type		All eukar
IPR013921	TATA-binding related factor		All eukar
IPR000007	Tubby, C-terminal		All eukar
IPR002653	Zinc finger, A20-type		All eukar
IPR000197	Zinc finger, TAZ-type		All eukar
IPR001766	Fork head transcription factor		All eukar

IPR000232	Heat shock factor (HSF)-type, DNA-binding	All eukar
IPR002100	Transcription factor, MADS-box	All eukar
IPR011598	Helix-loop-helix DNA-binding	All eukar
IPR004022	DDT	All eukar
IPR009395	GCN5-like 1	All eukar
IPR003316	Transcription factor E2F/dimerisation partner (TDP)	All eukar
IPR000818	TEA/ATTS	Metazoa+Fungi (some other eukar in trace amounts)
IPR024061	NDT80 DNA-binding domain	Fungi+Metazoa
IPR001356	Homeobox	Metazoa
IPR008967	p53-like transcription factor, DNA-binding	metazoa
IPR008917	Eukaryotic transcription factor, Skn-1-like	Metazoa
IPR015988	STAT transcription factor, coiled coil	Metazoa
IPR000418	Ets	Metazoa
IPR003902	Transcriptional regulator, GCM-like	metazoa
IPR001523	Paired box protein, N-terminal	Metazoa
IPR001275	DM DNA-binding	Metazoa
IPR010919	SAND-like	Metazoa
IPR001699	Transcription factor, T-box	Metazoa
IPR004826	Maf transcription factor	Metazoa
IPR000327	POU-specific	Metazoa
IPR006780	YABBY protein	Plants+Fungi
IPR004333	Transcription factor, SBP-box	Plants only
IPR003657	DNA-binding WRKY	Plant
IPR004645	DNA-binding protein Tfx	archea
IPR000944	Transcriptional regulator, Rrf2	Bacteria+archea
IPR010985	Ribbon-helix-helix	Bacteria+archea
IPR000005	Helix-turn-helix, AraC type	Bacteria
IPR010982	Lambda repressor-like, DNA-binding	Bacteria
IPR002197	Helix-turn-helix, Fis-type	Bacteria
IPR000792	Bacterial regulatory protein, LuxR	Bacteria
IPR000843	Bacterial regulatory protein, LacI	Bacteria
IPR016032	Signal transduction response regulator, C-terminal effector	Bacteria
IPR000835	Bacterial regulatory protein, MarR	Bacteria
IPR010921	Trp repressor/replication initiator	Bacteria
IPR001845	Bacterial regulatory protein, ArsR	Bacteria
IPR000551	Bacterial regulatory protein, MerR	Bacteria
IPR001034	Bacterial regulatory protein, DeoR N-terminal	Bacteria
IPR001808	Bacterial regulatory protein, Crp	Bacteria

* - in addition to fungi.
