# Supplementary material for: Transcription Factors in Fungi: TFome Dynamics, Three Major Families, and Dual-Specificity TFs
Source: Front Genet. 2017 May 4;8:53. doi: 10.3389/fgene.2017.00053 (PMC5415576; doi:10.3389/fgene.2017.00053)
Supplement: Table S5 — Responsiveness of fungal TFgFs to the proteome size growth. [file Table5.PDF]

Supplementary Material

Article Title Transcription factors in fungi: TFome dynamics, three major families, and dual-specificity TFs

Ekaterina Shelest*

*** Correspondence:** ekaterina.shelest@leibniz-hki.de

Table S5. Responsiveness of fungal TFgFs to the proteome size growth. **A.** Growth of TF gene families in Asco- and Basidiomycetes. The critical value for exponent (*exp*) and coefficient of determination (R^2) is 0,5: TFgFs that fulfill the requirement *exp*>0,5, R^2>0,5 are considered as growing. The analysis was made for TFs present in >10 genomes with >5 genes in at least one genome. Bold font, grey background: growing families; red: abundant families. **B.** Abundant families and how they grow.

A. TF family (>10 species):	*Ascomycetes*		*Basidiomycetes*	
	exp	R^2	exp	R^2
**Zn cluster**	**1,74**	**0,62**	**0,69**	**0,31**
**C2H2/CCHC/CCCH/C5HC2**	**0,88**	**0,83**	**0,88**	**0,67**
**HTH/Homeodomain-like**	**0,71**	**0,45**	**0,8**	**0,68**
**HLH, helix-loop-helix**	**0,66**	**0,45**	0,29	0,25
**bZIP**	**1,01**	**0,68**	0,19	0,09
Fork head TF	0,4	0,37	0,5	0,27
lambda repressor(-like)/POU	0,58	0,24	0,57	0,29
Zn cluster + C2H2/CCHC/CCCH-type ZF	1,49	0,25	0,19	0,02
Bacterial regulatory protein, LacI	0,09	0,01	0,13	0,01
Bacterial regulatory protein, LuxR	0,1	0	0,06	0,01
Bacterial regulatory protein, MarR	-0,03	0	-	-
bZIP + Helix-loop-helix DNA-binding	-0,11	0,04	0	1
bZIP+C2H2	0	1	-	-
C2H2/CCHC/CCCH ZF + Homeodomain	1,44	0,19	0,16	0,07
CBF/NF-Y/archaeal histone	0,07	0,02	0,21	0,2
CCAAT-BindingTF	0,2	0,1	0,09	0,04
Cold-shock DBD	-0,08	0	0,41	0,09
Copper fist	0,1	0,01	0,43	0,16
Copper fist+Zn finger	0	1	-0,07	0
DNA-binding, integrase-type	0	1	-0,44	0,2
DNA-binding, yeast	0,03	0	-0,08	0,02
GATA	0,12	0,02	0,3	0,23
GATA+Homeo	-	-	0	1
GCN5L1	0	1	-	-
Heat shock factor (HSF)-type	0,05	0,01	0,12	0,02
MADS-box/SRF	0,45	0,1	0,17	0,03
Mating-type protein MAT alpha 1	-0,01	0	-	-
p53	0,52	0,21	0,16	0,06
Putative DNA binding	0,21	0,05	0,47	0,16
RFX_DNA_binding	-0,12	0,1	0,06	0,04
Ribbon-helix-helix	0,04	0	0,36	0,14
SGT1	0	0	0	1
Signal transduction response regulator	-	-	0,38	0,27
Skn-1	0,08	0,04	0	1
ssDNA-binding transcriptional regulator	0,02	0	0,1	0,02
STAT	0,08	0	0,61	0,16
TATA-binding protein interacting (TIP20)	0	0	0,12	0,03
TATA-binding related factor	0	1	-	-
TEA/ATTS	0,02	0,01	0,69	0,22

TFIID	0,16	0,04	0,32	0,13
YL1	0	0	0,08	0,04
zf-BED	0,85	0,28	-	-
zf-GRF	0,32	0,07	0,16	0,02
zf-MIZ	0,34	0,15	0,06	0
Zn cluster + bZIP	0	1	-	-
Zn_cluster+C2H2+Homeodomain	0,02	0	-	-

B. Abundant families:

TF name	TFs per genome (average)	*Increases?*
Zn cluster	145	yes
C2H2/CCHC/CCCH/C5HC2	132	yes
HTH/Homeodomain-like	59	yes
bZIP	21	in ascomycetes
HLH, helix-loop-helix	13	in ascomycetes
GATA	10	no
CBF/NF-Y/archaeal histone	6	no
Fork head TF	5	(no)
Heat shock factor (HSF)-type	5	no

the rest of the TFgFs do not exceed 5 representatives per genome
