# Supplementary material for: Transcription Factors in Fungi: TFome Dynamics, Three Major Families, and Dual-Specificity TFs
Source: Front Genet. 2017 May 4;8:53. doi: 10.3389/fgene.2017.00053 (PMC5415576; doi:10.3389/fgene.2017.00053)
Supplement: Table S6 — List of proto- and metazoan genomes from DBD. [file Table6.PDF]

Supplementary Material

Article Title Transcription factors in fungi: TFome dynamics, three major families, and dual-specificity TFs

Ekaterina Shelest*

*** Correspondence:** ekaterina.shelest@leibniz-hki.de

Table S6. List of proto- and metazoan genomes from DBD (http://www.transcriptionfactor.org).

**METAZOA**
**Chordata**
Pan troglodytes (chimp)
Otolemur garnettii (galago)
Homo sapiens (human)
Macaca mulatta (macaca)
Microcebus murinus (mouse lemur)
Pongo abelii (orangutan)
Tupaia belangeri (tupaia)
Dasypus novemcinctus (armadillo)
Myotis lucifugus (bat)
Bos taurus (cow)
Felis catus (cat)
Canis familiaris (dog)
Loxodonta africana (elephant)
Ictidomys tridecemlineatus (ground squirrel)
Cavia porcellus (guinea pig)
Equus caballus (horse)
Erinaceus europaeus (hedgehog)
Mus musculus (mouse)
Monodelphis domestica (opossum)
Ochotona princeps (pika)
Ornithorhynchus anatinus (platypus)
Oryctolagus cuniculus (rabbit)
Rattus norvegicus (rat)
Sorex araneus (shrew)
Echinops telfairi (lesser hedgehog tenrec)
Danio rerio (zebrafish)
Gasterosteus aculeatus (three-spined stickleback)
Oryzias latipes (medaka)
Takifugu rubripes (takifugu)
Tetraodon nigroviridis (green spotted puffer)
Branchiostoma floridae (lancelet)
Gallus gallus (chicken)
Xenopus laevis (xenopus)
**Ecdysozoa (Arthropoda and Nematoda)**
Anopheles gambiae (anopheles)
Acyrthosiphon pisum (aphid)
Bombyx mori (silk moth)
Culex pipiens (southern house mosquito)

Drosophila melanogaster (drosophila)
Tribolium castaneum (flour_beetle)
Apis mellifera (honeybee)
Nasonia vitripennis (wasp)
Pediculus humanus (louse)
Aedes aegypti (yellow mosquito)
Daphnia pulex (daphnia)
Brugia malayi WS218 (nematode)
Caenorhabditis elegans
**PROTOZOA**
Cryptosporidium parvum Iowa II
Cryptosporidium hominis
Plasmodium falciparum 3D7
Plasmodium berghei ANKA
Plasmodium chabaudi
Plasmodium knowlesi strain H
Plasmodium yoelii ssp. yoelii 1
Phaeodactylum_tricornutum
Phytophthora infestans T30-4
Phytophthora ramorum 1.1
Phytophthora sojae
Thalassiosira_pseudonana
Theileria annulata
Theileria parva
Toxoplasma gondii RH
