# Supplementary material for: Transcription Factors in Fungi: TFome Dynamics, Three Major Families, and Dual-Specificity TFs
Source: Front Genet. 2017 May 4;8:53. doi: 10.3389/fgene.2017.00053 (PMC5415576; doi:10.3389/fgene.2017.00053)
Supplement: Table S8 — Responsiveness of metazoan TFgFs to the proteome size growth. [file Table8.PDF]

Supplementary Material

Article Title Transcription factors in fungi: TFome dynamics, three major families, and dual-specificity TFs

Ekaterina Shelest*

*** Correspondence:** ekaterina.shelest@leibniz-hki.de

Table S8. Responsiveness of metazoan TFgFs to the proteome size growth. **A.** Characteristics of TF gene families' expansion behavior in chordates and ecdysozoans (arthropods + nematodes). The critical value for the exponent (*exp*) and coefficient of determination (R^2) is 0,5. The second column shows the abundance of a family in vertebrates (v) or invertebrates (i). Grey background: expanding families; in parentheses: the number of considered species.

		chordates (33)		ecdysozoans (13)	
TF gene family	***abundant in***	***exp***	***R^2***	***exp***	***R^2***
HTH/Homeodomain-like	i,v	0,96	0,56	0,46	0,59
C2H2/CCHC/C5HC2	i,v	1,08	0,5	0,47	0,15
HLH, helix-loop-helix	i,v	1,19	0,64	0,26	0,21
Fork head TF	i,v	1,29	0,54	0,11	0,06
IRF	v	1,17	0,61	-	-
bZIP	i,v	1,02	0,51	0,24	0,18
Myogenic Basic domain		0,89	0,47	-	-
zf-BED	i	1,76	0,27	1,74	0,54
DNA-binding domain	v	0,26	0,08	1,22	0,6
GR-like	i,v	0,7	0,44	0,36	0,03
A DBD in eukar_TF		0,43	0,07	-	-
AF-4		-0,38	0,15	-	-
AP-2		0,14	0,01	-	-
BESS	v	-	-	1,49	0,18
bZIP+C2H2		0,29	0,02	-	-
C2H2/CCHC/CCCH ZF + Homeodomain		0,87	0,3	-0,54	0,22
C2H2+Glucocorticoid receptor-like	i	-	-	-1,06	0,07
CG-1		0,39	0,06	-	-
Cold-shock DBD	v	0,29	0,08	0,22	0,06
CUT, Homeodomain+CUT	v	0,27	0,04	0,13	0,02
DM DNA-binding		1,23	0,4	0,63	0,2
E2F_TDP	v	0,29	0,08	-	-
Ets	i,v	0,27	0,16	0,32	0,38

Fez1		-0,12	0,01	-	-
GATA		0,23	0,02	-	-
GR-like+GATA	i,v	0,83	0,28	0,44	0,15
Heat shock factor (HSF)-type		0,71	0,17		
Homeodomain+GR-like	i,v	0,76	0,19	-0,13	0,01
Homeodomain+lambda repressor-like		1,35	0,39	-	-
lambda repressor/POU	v	0,71	0,23	-0,18	0,11
lambda repressor-like		0,62	0,09	0,22	0,02
MADS-box/SRF		0,79	0,3	-	-
Myc_N		0,22	0,02	-	-
p53	i,v	0,47	0,28	0,24	0,08
RFX_DNA_binding	v	0,5	0,15	-	-
SAND-like		0,82	0,22	-	-
SMAD MH1 domain	v	0,66	0,28	0,22	0,06
STAT	v	0,03	0	-	-
TEA/ATTS		-0,37	0,08	-	-
Tubby TF		-0,07	0	-	-
Winged helix DNA-binding		1,02	0,12	-	-
zf-A20		0,79	0,27	-	-
zf-GRF		0,36	0,12	-	-
zf-MIZ		0,4	0,06	0	1
zf-TAZ		0,98	0,41	0,59	0,15
